# A Multi-Criteria Decision Support Framework for Prioritizing the Repair of Failed Medical Devices: A Hospital Case Study

**DOI:** 10.3390/healthcare14172799

**Published:** 2026-09-01

**Authors:** Elif Tarakçı, Recep Duranay, Muhammed Reşit Kaplan

**Affiliations:** 1 Department of Industrial Engineering, Faculty of Engineering and Natural Sciences, Istanbul Atlas University, Hamidiye, Anadolu Cd. no:40, 34408 Istanbul, Türkiye; 2 Department of Computer Engineering, Faculty of Engineering and Natural Sciences, Istanbul Atlas University, Hamidiye, Anadolu Cd. no:40, 34408 Istanbul, Türkiye; recep.duranay@atlas.edu.tr (R.D.); resit.kaplan@atlas.edu.tr (M.R.K.)

**Keywords:** medical device repair prioritization, healthcare technology management, decision support system, patient safety, AHP, TOPSIS, fuzzy TOPSIS, multi-criteria decision making

## Abstract

**Background/Objectives:** The delayed repair of failed medical devices, particularly when multiple devices are simultaneously unavailable, may adversely affect patient safety and healthcare continuity, making repair prioritization a critical hospital decision problem. This study develops an integrated multi-criteria decision-making (MCDM) framework for prioritizing failed medical devices for repair and evaluates the consistency and robustness of the resulting priorities. Its principal contribution is to address repair prioritization as a distinct operational decision problem by integrating deterministic and fuzzy MCDM approaches and robustness analyses within a unified framework. **Methods:** Seven evaluation criteria—patient safety risk (PSR), device function (DF), device criticality (DC), utilization frequency (UF), availability of alternative devices (AAD), resource impact (RSI), and device age (DA)—were considered. The framework was implemented in a hospital case study involving 32 medical devices using the Analytic Hierarchy Process (AHP), AHP-weighted scoring, the Technique for Order Preference by Similarity to Ideal Solution (TOPSIS), Fuzzy Analytic Hierarchy Process (Fuzzy AHP), and Fuzzy Technique for Order Preference by Similarity to Ideal Solution (Fuzzy TOPSIS). Robustness was examined through ±10%, ±30%, and ±50% criterion-weight sensitivity analyses, criterion-removal analysis, comparative ranking analysis, and hospital expert-based AHP and class-based AHP scenarios. **Results:** In the baseline AHP-weighted scoring ranking, Anesthesia Machine (4.9233) and External Cardiac Pacemaker (4.8862) received the highest repair priorities. Comparisons with TOPSIS, Fuzzy TOPSIS, hospital expert-based AHP, and class-based AHP yielded Spearman’s rank correlation coefficient (ρ) values ranging from 0.8328 to 0.9952. Sensitivity analysis showed high overall ranking stability under substantial criterion-weight variations, while criterion-removal analysis also maintained high correlations (ρ = 0.9901–0.9963), supporting the robustness of the overall ranking structure. **Conclusions:** The findings demonstrate that the framework can support hospital biomedical units in prioritizing failed devices, enhance the transparency and consistency of repair decisions, and contribute to more effective maintenance resource allocation.

## 1. Introduction

Technological advancements have increased hospitals’ dependence on sophisticated medical devices, making their safe, reliable, and continuous operation essential for patient care and operational efficiency. However, underutilization, maintenance disruptions, and fluctuations in device performance can adversely affect both healthcare delivery and hospital operations [1]. Inadequate technical support, limited spare-part availability, and insufficient user training may further prolong device unavailability and compromise operational continuity [2]. Medical equipment maintenance is generally categorized as preventive or corrective maintenance. While preventive maintenance aims to reduce the likelihood of failure through planned interventions, corrective maintenance is initiated after a failure has occurred and may therefore result in equipment downtime and increased patient-safety and operational risks [3]. As medical devices become increasingly complex, effective maintenance management is consequently essential to ensuring their required performance and availability [4]. In corrective maintenance settings, this challenge becomes particularly important when multiple failed devices compete for limited maintenance resources, requiring the biomedical unit to determine which device should receive repair intervention first.

Recent research on medical device maintenance has increasingly focused on data-driven, predictive, and technology-supported approaches. Machine-learning models have been developed to predict medical device failures using structured and unstructured maintenance data [5], while alternative validation strategies have been investigated to improve the reliability of predictive maintenance models under real hospital operating conditions [6]. Other studies have examined the financial performance of internal and outsourced maintenance structures [7], technology-enabled monitoring of device reprocessing cycles to support proactive maintenance [8], and information systems for organizing device records, maintenance histories, protocols, and repair activities [9]. Collectively, these studies have contributed to improving failure prediction, maintenance planning, monitoring, and maintenance management.

MCDM and fuzzy decision-making approaches have also been widely applied to healthcare problems requiring the simultaneous evaluation of multiple criteria and uncertainty. Previous applications include an intuitionistic fuzzy AHP–TOPSIS model for healthcare waste treatment selection [10], a fuzzy decision-support system for hospital service-quality improvement [11], and fuzzy TOPSIS for prioritizing enabling technologies for Quality 4.0 implementation in healthcare [12]. These studies illustrate the applicability of different multi-criteria and fuzzy decision-support approaches across diverse healthcare decision contexts. More specifically, within health technology and medical device management, MCDM approaches have been used for medical device selection under uncertainty [13], assessment of the maintenance condition of medical ventilators [14], selection of healthcare management systems under resource constraints [15], and prioritization of maintenance-related failure modes considering risk and cost [16]. Collectively, these studies demonstrate the value of structured multi-criteria approaches for healthcare technology and maintenance decisions.

Despite these advances, the prioritization of failed medical devices for repair remains comparatively underexplored as a distinct operational decision problem. When multiple devices are simultaneously unavailable, biomedical units must determine which failed device should be repaired first. This decision differs from preventive and predictive maintenance planning, replacement decisions, general device criticality assessment, and failure-mode prioritization because it requires the urgency of restoring each failed device to clinical service to be evaluated from multiple clinical and operational perspectives. Although previous studies provide valuable approaches to medical device maintenance and prioritization, comparatively limited attention has been given to directly ranking failed medical devices by simultaneously considering PSR, DF, DC, UF, AAD, RSI, and DA. The principal original contribution of this study is therefore that, unlike previous studies that have predominantly focused on failure prediction, preventive maintenance planning, device criticality assessment, and the prioritization of maintenance-related failure modes, it addresses the decision of which medical device should be repaired first and returned to clinical service when multiple devices have failed, considering the potential adverse consequences of repair delays for patient safety, continuity of clinical services, hospital operations, and resource utilization, as a distinct operational decision problem. To address this problem, the study develops an MCDM-based decision-support framework that integrates PSR, DF, DC, UF, AAD, RSI, and DA based on the assessments of experienced experts with knowledge of hospital and biomedical maintenance processes. The framework further enables the robustness of the resulting priorities to be examined through deterministic and fuzzy MCDM approaches, sensitivity and criterion-removal analyses, and comparisons with hospital expert- and class-based prioritization.

Accordingly, this study aims to develop and evaluate a structured decision-support framework for prioritizing failed medical devices for repair in hospital environments. Specifically, the study determines criterion weights using AHP, generates and compares device rankings through AHP-weighted scoring, TOPSIS, and fuzzy MCDM approaches, and evaluates the robustness of the resulting priorities through sensitivity, criterion-removal, and comparative ranking analyses. The resulting rankings are further examined using a hospital expert-based AHP scenario and a class-based AHP scenario representing the case hospital’s class-based prioritization logic. Through this design, the study evaluates the consistency and robustness of the proposed framework and examines how alternative methodological and operational assumptions affect device rankings.

The remainder of this paper is organized as follows. Section 2 describes the materials, evaluation criteria, and decision-making methods employed in the study. Section 3 presents the repair prioritization results, sensitivity and criterion-removal analyses, comparative ranking analyses, and the hospital expert-based AHP and class-based AHP scenarios. Section 4 discusses the findings, highlights their practical implications, and addresses the study limitations and directions for future research. Finally, Section 5 summarizes the main conclusions of the study.

## 2. Materials and Methods

This section presents the methodology of the proposed medical device repair prioritization framework. First, the overall framework, evaluation criteria, data collection process, and decision structure are described. Subsequently, the AHP-based weighted scoring and TOPSIS procedures used to prioritize the medical devices are presented. For the fuzzy analysis, Fuzzy AHP is employed to derive the criterion weights, which are subsequently incorporated into Fuzzy TOPSIS to obtain the final device ranking. Finally, the sensitivity analyses, criterion-removal analyses, alternative prioritization scenarios, and comparative ranking analysis used to assess the robustness and consistency of the proposed framework are described.

### 2.1. Framework of Proposed Model

The proposed framework was developed to support the systematic prioritization of failed medical devices requiring corrective repair when multiple devices compete for limited maintenance resources. The framework integrates expert knowledge, hospital-specific information, and multi-criteria decision-making methods to generate and compare repair priority rankings.

The framework begins with the identification of the evaluation criteria and medical devices included in the analysis. The criteria and devices were determined based on literature and expert assessment, and the devices were evaluated using a common five-point scoring scale. The three experts jointly assessed the pairwise comparisons and device-level criterion scores and reached consensus, resulting in a single common pairwise comparison matrix and a common decision matrix used in the main analysis.

**Figure 1:**
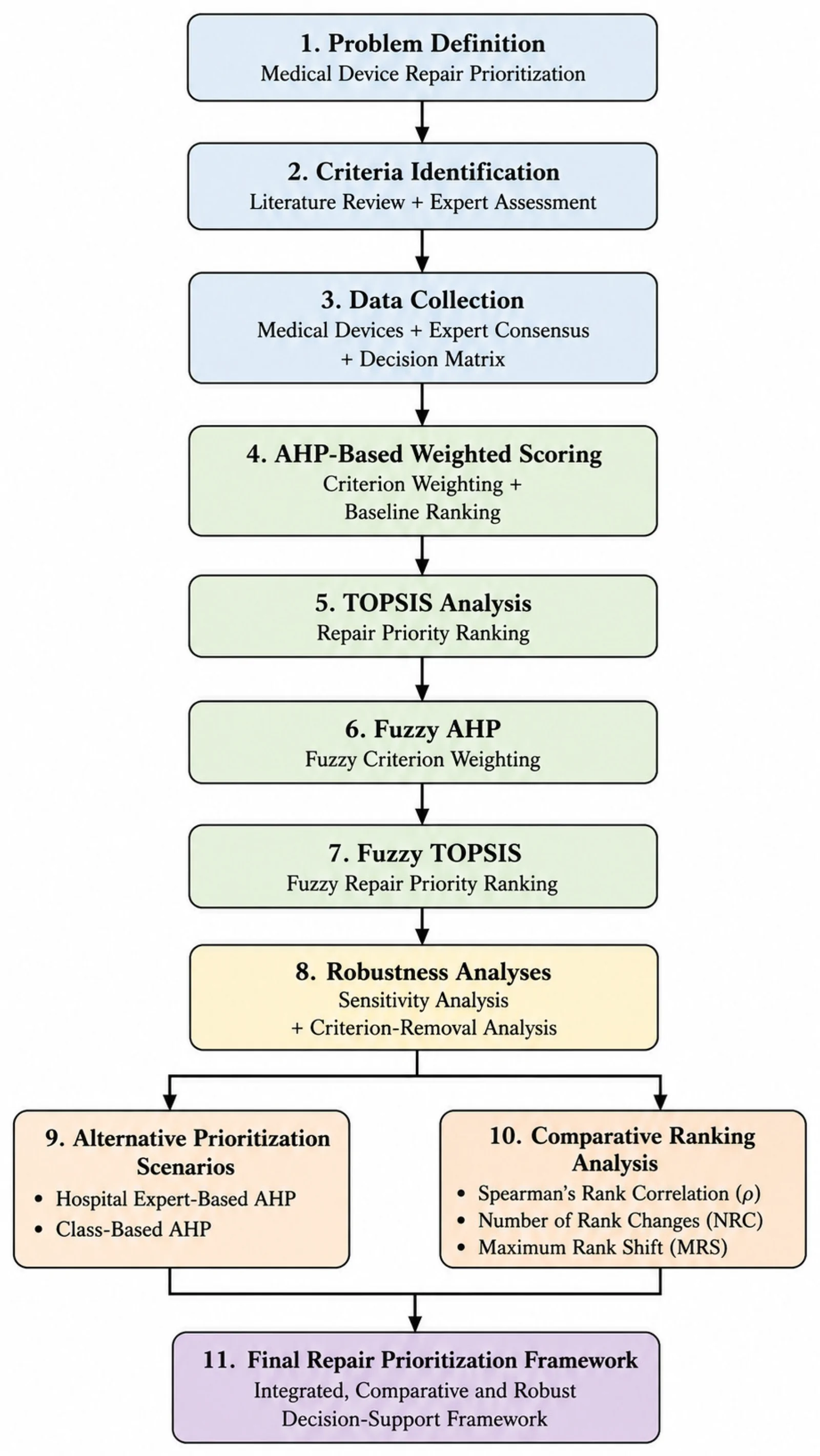
Proposed research framework for medical device repair prioritization.

The main deterministic analysis consists of two complementary approaches. First, AHP is used to derive the relative weights of the evaluation criteria, while the medical devices are ranked using an AHP-based weighted scoring model. Second, the same AHP-derived criterion weights and decision matrix are incorporated into TOPSIS to obtain an alternative ranking based on relative closeness to the ideal solution. In the fuzzy analysis, the consensus pairwise comparison judgments are represented by triangular fuzzy numbers (TFNs), and Fuzzy AHP is used to derive fuzzy criterion weights. These weights are subsequently incorporated into Fuzzy TOPSIS, which produces the final fuzzy repair priority ranking of the medical devices. The robustness of the main prioritization results is examined through one-at-a-time sensitivity analyses in which criterion weights are varied by ±10%, ±30%, and ±50%. In addition, criterion-removal analyses are performed for PSR, DF, and DC to examine whether their conceptual proximity materially affects the resulting ranking. Two structured comparison scenarios, hospital expert-based AHP and class-based AHP, are also evaluated to examine how the proposed consensus-based framework compares with alternative expert- and class-based prioritization structures. Finally, the rankings obtained from the different approaches are compared using Spearman’s rank correlation coefficient, the Number of Rank Changes (NRC), and the Maximum Rank Shift (MRS), and the most affected devices. The overall structure of the proposed framework is illustrated in Figure 1.

### 2.2. Criteria Identification

To systematically prioritize the repair of failed medical devices, it is first necessary to identify the criteria influencing the decision-making process. Accordingly, a comprehensive literature review covering medical device maintenance management, medical device criticality assessment, healthcare technology management, and applications of MCDM was conducted to identify the relevant evaluation criteria. The findings obtained from the literature were subsequently supported and refined through expert opinions, and the evaluation criteria used in this study were established.

Taghipour et al. [17] proposed an Analytic Hierarchy Process (AHP)-based multi-criteria decision-making model to support maintenance prioritization of medical devices. The model consists of 16 criteria and sub-criteria organized within a hierarchical structure for assessing medical device criticality. The hierarchy comprises six main criteria: function (C1), mission criticality (C2), age (C3), risk (C4), recalls and hazard alerts (C5), and maintenance requirements (C6). These main criteria are further supported by sub-criteria such as utilization rate, availability of alternative devices, failure frequency, detectability, and failure consequences. According to the authors, the proposed framework enables hospitals to allocate maintenance resources more effectively and to identify the most appropriate maintenance strategy for each category of medical devices. Jamshidi et al. [18] proposed a fuzzy multi-criteria decision-making framework for medical device prioritization. The model combines Fuzzy Failure Mode and Effects Analysis with additional evaluation dimensions, including age, usage-related hazards, utilization rate, availability of alternative devices, recalls, function, and maintenance requirements, to provide a comprehensive assessment of medical device risk. Saleh et al. [19] developed a Quality Function Deployment-based model to prioritize preventive maintenance activities. Their framework incorporates 11 critical factors grouped into three categories: risk-based criteria (e.g., function, physical risk, and maintenance requirements), mission-based criteria (e.g., utilization level, area criticality, and device criticality), and maintenance-based criteria (e.g., failure rate, service life ratio, maintenance complexity, missed maintenance, and downtime). Zamzam et al. [20] proposed a machine learning-based approach for medical device maintenance and replacement planning using 16 device-related attributes, including age, support service, asset condition, function, preventive maintenance status, maintenance status, number of missed planned preventive maintenance, request-to-repair time, maintenance requirement, maintenance complexity, repair time, downtime, number of failures, asset status, and backup or alternative unit operations. Liao et al. [21] developed a Support Vector Machine model to predict repair requirements based on failure frequency, time between failures, and time to repair. Abd Rahman et al. [22] also applied machine learning and deep learning techniques to predict medical device failures using variables such as age, service support, asset condition, maintenance complexity, total downtime, total maintenance cost, device model, and purchase date. Hutagalung and Hasibuan [23] proposed an AHP-based maintenance prioritization model using seven main criteria, including degree of complexity of the maintenance, function, risk, degree of importance of mission, age, recall and user errors, and medical equipment class. Shamayleh et al. [24] adopted an Internet of Things-based predictive maintenance approach, employing vibration signal features such as kurtosis, peak-to-peak value, standard deviation, and root mean square to detect mechanical faults in biochemical analyzers. Beniacoub et al. [25] developed a prioritization index incorporating equipment type, device function, maintenance and calibration requirements, age, location, and risk, while additionally classifying devices according to their operational condition and technical criticality.

Based on the criteria identified in the literature, an initial pool of potentially relevant criteria was established. These criteria were subsequently reviewed by three experts and one hospital expert with respect to their relevance and applicability to the specific operational problem of corrective repair prioritization. Through joint expert evaluation, the candidate criteria were reviewed and refined, and consensus was reached on a final set of seven criteria: PSR, DF, DC, UF, AAD, RSI, and DA. Although the literature includes additional factors such as failure frequency, maintenance complexity, downtime, maintenance cost, recalls, and replacement-related indicators, not all of these were retained as separate criteria. The present study specifically focuses on determining the priority of repair intervention after a device failure has occurred, rather than predicting future failures, planning preventive maintenance, or supporting equipment replacement decisions. Accordingly, the final criteria were selected to capture the clinical, operational, and resource-related dimensions directly relevant to the urgency of corrective repair. The definitions and operational interpretations of the seven criteria used in the proposed framework are presented in Table 1.

Particular attention was given to the conceptual distinction among PSR, DF, and DC, since these criteria are clinically related but represent different dimensions of repair prioritization. PSR represents the potential patient-level consequences associated with device failure and therefore reflects the severity of the failure consequence from a patient-safety perspective. DF describes the intrinsic and relatively stable medical function of the device, ranging from supportive to life-support functions. In contrast, DC represents the context-dependent importance and indispensability of the device within prevailing clinical care processes. Thus, PSR, DF, and DC, respectively, capture failure consequence, intrinsic device function, and context-dependent clinical criticality, rather than measuring the same construct. To further assess whether their conceptual relatedness materially influenced the prioritization results, an additional criterion-removal robustness analysis was conducted in which PSR, DF, and DC were individually excluded and the resulting rankings were compared with the baseline ranking.

For operational evaluation, all seven criteria were defined using a five-point scoring scale, with their criterion-specific score levels presented in Table 2. The criteria were oriented in a common preferential direction so that a higher score consistently represented a greater need for timely repair intervention. For PSR, DF, DC, and UF, higher scores indicate greater patient-safety risk, functional importance, clinical criticality, and utilization intensity, respectively. For AAD, the scoring scale was inversely structured with respect to the availability of substitutes: a higher score indicates lower availability of suitable alternative devices and therefore a greater need for prompt restoration of the failed device. RSI was defined as the broader operational and resource pressure generated by device failure, rather than repair cost alone. Accordingly, a higher RSI score represents greater disruption or pressure involving workforce, clinical and maintenance scheduling, technical resources, and budget. DA was operationalized as an age-related maintenance requirement, rather than assuming that an older device should automatically receive a higher repair priority. A higher DA score therefore reflects the increased need for timely diagnostic and technical assessment and the potential for more complex maintenance intervention as device age increases. These definitions and scoring directions ensure that the numerical scores in Table 2 have a consistent interpretation within the repair-prioritization framework.

### 2.3. Data Collection

The study was conducted in a university hospital located in Istanbul, Türkiye. The hospital has approximately 400 beds, a closed area of approximately 75,000 m^2^, 19 operating rooms, approximately 100 intensive care beds/units, approximately 120 physicians, and a total workforce of approximately 1300 personnel. Its biomedical inventory comprises approximately 3000 medical devices. The hospital provides services across operating rooms, intensive care units, emergency services, cardiology, radiological imaging, laboratories, sterilization, neonatal care, and other diagnostic and therapeutic units. This large-scale and multidisciplinary structure results in the simultaneous management of medical devices with substantially different clinical functions, criticality levels, utilization patterns, and maintenance requirements. The hospital was therefore considered an appropriate case setting for examining the prioritization of corrective repair activities under limited biomedical maintenance resources.

The biomedical unit is responsible for hospital-wide medical device inventory management, receipt and monitoring of failure notifications, creation and follow-up of work orders, coordination of corrective maintenance activities and external technical services when required, and technical and operational monitoring of medical devices. Since January 2024, failure notifications, work orders, and associated maintenance activities have been recorded through a digital biomedical maintenance tracking application. Accordingly, the operational information used to support the present case study covered the period from January 2024 to April 2026. Device-related information and expert assessments were developed with reference to the inventory information, maintenance records, work orders, and operational experience accumulated during this observation period. Device age was determined using commissioning/purchase information available in the hospital inventory records, with the study data cut-off date used as the reference point. Regarding the existing repair prioritization practice, medical devices at the hospital are categorized according to predefined criticality classes (Class 1, Class 2, and Class 3). When multiple failed devices belong to the same criticality class, repair priorities are determined based on the experience of biomedical maintenance personnel and prevailing operational conditions rather than through a formal multi-criteria decision-making procedure. Factors such as the availability of alternative devices, the need for external technical service, expected repair time, and current clinical requirements may be considered on a case-by-case basis. Thus, the existing hospital practice represents an experience-based and operationally adaptive prioritization process rather than a structured multi-criteria evaluation framework.

The medical devices included in the analysis were selected from the hospital inventory rather than being treated as a set of 32 devices that had failed simultaneously. The Proposal of the initial medical device set was proposed by the case-hospital expert on the basis of the hospital’s biomedical inventory, maintenance experience, clinical relevance, and the operational consequences associated with device unavailability. The suitability of the proposed devices for the corrective repair prioritization problem was subsequently reviewed by the other three experts. Following this review and the exclusion of items that did not meet the final scope of the analysis, 32 medical devices were retained. These devices cover different levels of clinical criticality and a broad range of diagnostic, therapeutic, life-support, monitoring, laboratory, and sterilization functions. The devices and their hospital criticality classes are presented in Table 3. To provide further context regarding the heterogeneity of the analyzed equipment, their basic clinical use, typical clinical unit, and significance of failure are reported in Supplementary Table S1.

Expert judgment was incorporated at several stages of the study. The expert panel consisted of three professionals with biomedical engineering, biomedical device management, and medical device maintenance experience, together with one biomedical device technician from the case hospital with 12 years of professional experience. The professional backgrounds, experience, expertise, and specific roles of the experts are summarized in Table 4. The experts contributed to the refinement of the evaluation criteria and jointly evaluated the 32 medical devices according to the seven criteria using the predefined 1–5 scoring scale presented in Table 2. For the main model, the AHP pairwise comparison matrix used to derive criterion weights was likewise established through expert consensus among E1–E3. The hospital expert additionally provided the pairwise comparison judgments used in the hospital expert-based AHP and class-based AHP scenarios, which were constructed as alternative representations of hospital-oriented prioritization logic. The resulting 32 × 7 decision matrix therefore represents a structured expert assessment informed by the hospital’s inventory, maintenance records, work-order information, and operational experience during the observation period. The matrix should not be interpreted as an observed chronological repair sequence or as a set of 32 simultaneous failures. Although historical failure notifications and work-order records are available in the hospital’s digital maintenance system, an actual observed hospital repair order was not used as an external validation benchmark in the present analysis. Accordingly, the hospital expert-based AHP and class-based AHP analyses were treated as alternative prioritization scenarios rather than empirical validation against actual repair decisions.

### 2.4. AHP Method and AHP-Weighted Scoring

AHP, developed by Thomas L. Saaty, is one of the most widely used MCDM methods for determining criterion weights [26]. Accordingly, AHP was employed in this study to calculate the relative importance of the evaluation criteria. First, the decision problem was structured hierarchically, and the evaluation criteria were compared pairwise using Saaty’s 1–9 fundamental scale. The resulting pairwise comparison matrix was then normalized to derive the criterion weights. The consistency of the pairwise comparisons was evaluated using the Consistency Index (CI) and the Consistency Ratio (CR), and the comparisons were considered acceptable when CR < 0.10. Saaty’s fundamental scale and the Random Index (RI) values used in the AHP analysis are presented in Table 5 and Table 6, respectively, while the equations used for the consistency calculations are given in Equations (1) and (2) [27].

(1) $$CI=\frac{\lambda_{\max}-n}{n-1}$$

(2) $$CR=\frac{CI}{RI}$$

Each medical device was evaluated against the selected criteria using a five-point scoring scale, and the corresponding decision matrix was constructed. The decision matrix is represented by X = [$x_{ij}$], where $x_{ij}$ denotes the score assigned to the $i^{th}$ medical device with respect to the $j^{th}$ evaluation criterion, as shown in Equation (3). The five-point ordinal scale was used as a structured scoring convention to translate expert assessments into comparable priority levels. For the AHP-weighted scoring procedure, these numerical scores were treated as operational approximations of increasing priority intensity to enable weighted aggregation, rather than as interval-scale measurements for statistical inference. The robustness of the resulting rankings was subsequently examined through criterion-weight sensitivity and criterion-removal analyses. The resulting decision matrix was subsequently used in both the AHP-based weighted scoring approach and the TOPSIS analysis.

(3) $$P_{i}=\sum_{j=1}^{n}w_{j}x_{ij}$$

### 2.5. TOPSIS Method

In this study, TOPSIS was employed to prioritize the repair of failed medical devices. Developed by Hwang and Yoon, TOPSIS is one of the most widely used MCDM methods, which ranks alternatives according to their relative closeness to the positive ideal solution (PIS) and their distance from the negative ideal solution (NIS) [28]. The common decision matrix constructed from the expert evaluations was subsequently used in the TOPSIS analysis. First, the decision matrix was normalized to obtain the normalized decision matrix, thereby making the criterion values comparable. Subsequently, the weighted normalized decision matrix was constructed using the criterion weights derived from the AHP analysis. Next, the PIS and NIS were determined, and the separation distances of each alternative from these ideal solutions were calculated. Finally, the relative closeness coefficients (CC) were computed, and the medical devices were ranked accordingly. A higher CC indicates that a medical device is closer to the positive ideal solution and therefore has a higher repair priority. The main computational steps of the TOPSIS method are presented in Equations (4)–(8) [28].

(4) $$r_{ij}=\frac{x_{ij}}{\sqrt{\sum_{i=1}^{m}x_{ij}^{2}}}$$

(5) $$v_{ij}=w_{j}r_{ij}$$

(6) $$A^{+}=\{v_{1}^{+},v_{2}^{+},\ldots ,v_{n}^{+}\}$$

(7) $$A^{-}=\{v_{1}^{-},v_{2}^{-},\ldots ,v_{n}^{-}\}$$

(8) $$CC_{i}=\frac{S_{i}^{-}}{S_{i}^{+}+S_{i}^{-}}$$

In these equations, $r_{ij}$ denotes the elements of the normalized decision matrix, $w_{j}$ represents the weight of the $j^{th}$ criterion, $v_{ij}$ denotes the elements of the weighted normalized decision matrix, $A^{+}$ and $A^{-}$ represent the positive and negative ideal solutions, respectively, $S_{i}^{+}$ and $S_{i}^{-}$ denote the separation distances of alternative i from the positive and negative ideal solutions, respectively, and $CC_{i}$ represents the relative CC [28].

### 2.6. Integrated Fuzzy AHP–Fuzzy TOPSIS Method

Conventional AHP and TOPSIS require decision makers to express their judgments using precise numerical values. However, in real-world decision-making processes, expert judgments often involve uncertainty and hesitation. To address this limitation, the present study employed the Fuzzy AHP and Fuzzy TOPSIS methods based on the fuzzy set theory proposed by Zadeh [29]. Fuzzy set theory enables uncertain and imprecise expert judgments to be represented using TFNs. A triangular fuzzy number is expressed as:

(9) $$\tilde{A}=(l,m,u)$$

In Equation (9), l, m, and u represent the lower limit, the most probable value, and the upper limit, respectively [29].

The pairwise comparison judgments expressed using Saaty’s 1–9 scale were converted into TFNs according to the fuzzy conversion scale presented in Table 7.

The three experts jointly evaluated the pairwise comparisons and reached consensus, resulting in a single common pairwise comparison matrix. This consensus pairwise comparison matrix was subsequently converted into TFNs using the predefined fuzzy scale. Thus, the fuzzy analysis was based on the same consensus judgments used for criterion weighting in the main model.

The Fuzzy AHP procedure based on Buckley’s geometric mean method [30] was used to calculate the fuzzy criterion weights. The main computational steps of the integrated Fuzzy AHP–Fuzzy TOPSIS procedure are presented in Equations (10)–(20).

For each criterion, the fuzzy geometric mean of the corresponding row was calculated as:

(10) $${\tilde{r}}_{i}={\left(\prod_{j=1}^{n}{\tilde{a}}_{ij}\right)}^{1/n}=\left(l_{i},m_{i},u_{i}\right)$$

The fuzzy weight of criterion was then determined as:

(11) $${\tilde{w}}_{i}={\tilde{r}}_{i}⊗{\left(\sum_{i=1}^{n}{\tilde{r}}_{i}\right)}^{-1}=\left(l_{wi},m_{wi},u_{wi}\right)$$

where ${\tilde{a}}_{ij}$ represents the fuzzy pairwise comparison value, ${\tilde{r}}_{i}$ denotes the fuzzy geometric mean, ${\tilde{w}}_{i}$ represents the fuzzy weight of criterion i.

For reporting and interpretation, the fuzzy criterion weights were converted into crisp values using the Center of Area (COA) method:

(12) $$w_{i}^{crisp}=\frac{l_{wi}+m_{wi}+u_{wi}}{3}$$

The resulting crisp weights were subsequently normalized so that their sum was equal to 1. The final fuzzy weights and normalized crisp criterion weights are reported in Section 3. The fuzzy weights, rather than the defuzzified crisp weights, were subsequently used in the Fuzzy TOPSIS weighting procedure. The Fuzzy TOPSIS procedure proposed by Chen [31] was applied to rank the medical devices using the fuzzy criterion weights obtained from Fuzzy AHP.

The common 32 × 7 decision matrix contained criterion scores ranging from 1 to 5 according to the evaluation scale presented in Table 2. For the Fuzzy TOPSIS analysis, the crisp decision matrix was transformed into a fuzzy decision matrix using TFNs. The resulting fuzzy decision matrix was subsequently normalized and weighted using the fuzzy criterion weights obtained from the Fuzzy AHP analysis.

Since all criteria were oriented so that higher values corresponded to greater repair priority, they were treated as benefit criteria. The fuzzy decision matrix was normalized using the maximum upper-bound value for each criterion:

(13) $${\tilde{r}}_{ij}=\left(\frac{a_{ij}}{u_{j}^{*}},\frac{b_{ij}}{u_{j}^{*}},\frac{c_{ij}}{u_{j}^{*}}\right)$$

where:

(14) $$u_{j}^{*}=\underset{i}{\max}c_{ij}$$

and $a_{ij},\;b_{ij},\;c_{ij}$ denotes the TFN corresponding to alternative i under criterion j.

The normalized fuzzy decision values were multiplied by the fuzzy criterion weights obtained from Fuzzy AHP:

(15) $${\tilde{v}}_{ij}={\tilde{r}}_{ij}⊗{\tilde{w}}_{j}$$

For triangular fuzzy numbers, this multiplication was implemented component-wise as:

(16) $${\tilde{v}}_{ij}=\left(l_{ij}\cdot l_{w_{j}},m_{ij}\cdot m_{w_{j}},u_{ij}\cdot u_{w_{j}}\right)$$

For each criterion, the Fuzzy Positive Ideal Solution (FPIS) and Fuzzy Negative Ideal Solution (FNIS) were determined from the weighted normalized fuzzy decision matrix. The FPIS was defined using the maximum upper bound for each criterion, whereas the FNIS was defined using the minimum lower bound.

The distance between two triangular fuzzy numbers,

${\tilde{m}}_{1}=\left(l_{1},m_{1},u_{1}\right)$ and ${\tilde{m}}_{2}=\left(l_{2},m_{2},u_{2}\right)$ was calculated using:

(17) $$d\left({\tilde{m}}_{1},{\tilde{m}}_{2}\right)=\sqrt{\frac{1}{3}\left[{\left(l_{1}-l_{2}\right)}^{2}+{\left(m_{1}-m_{2}\right)}^{2}+{\left(u_{1}-u_{2}\right)}^{2}\right]}$$

The total distances of each medical device from the FPIS and FNIS were then calculated by summing the criterion-specific distances:

(18) $$d_{i}^{*}=\sum_{j=1}^{n}d\left({\tilde{v}}_{ij},{\tilde{v}}_{j}^{*}\right)$$

(19) $$d_{i}^{-}=\sum_{j=1}^{n}d\left({\tilde{v}}_{ij},{\tilde{v}}_{j}^{-}\right)$$

The closeness coefficient of each medical device was calculated as:

(20) $${\mathrm{CC}}_{i}=\frac{d_{i}^{-}}{d_{i}^{*}+d_{i}^{-}},\;\;0\leq {\mathrm{CC}}_{i}\leq 1$$

A higher ${\mathrm{CC}}_{i}$ indicates that the device is closer to the fuzzy positive ideal solution and therefore has a higher repair priority. The medical devices were ranked in descending order according to their ${\mathrm{CC}}_{i}$ values. Devices with identical ${\mathrm{CC}}_{i}$ values were considered to have equal priority according to Fuzzy TOPSIS. For sequential presentation in the results table, the display order of tied devices was determined based on expert judgment; however, this ordering was not interpreted as a methodological difference in priority between the tied devices. The integrated Fuzzy AHP–Fuzzy TOPSIS analysis was implemented in MATLAB R2024a (MathWorks, Natick, MA, USA). The complete MATLAB codes used to reproduce the Fuzzy AHP criterion-weight calculations, and the Fuzzy TOPSIS device-ranking procedure are provided in the Supplementary Materials.

### 2.7. Sensitivity Analysis Method

A sensitivity analysis was conducted to evaluate the robustness of the proposed decision model against variations in the criterion weights. For this purpose, the weight of each of the seven evaluation criteria was individually varied by ±10%, ±30%, and ±50% relative to its baseline value. Following each modification, the criterion weights were re-normalized to ensure that their sum remained equal to one. Considering three variation levels in both positive and negative directions for each of the seven criteria, a total of 42 weight-variation scenarios were analyzed. For each scenario, the AHP-weighted scoring results and TOPSIS rankings were recalculated and compared with the corresponding baseline rankings. This analysis enabled the stability of the repair priority rankings to be evaluated under progressively larger changes in individual criterion weights.

### 2.8. Criterion Removal Analysis

To further examine the robustness of the proposed framework and its dependence on the most influential criteria, a criterion-removal analysis was conducted for the three highest-weighted criteria in the baseline model: PSR, DF, and DC. These criteria were selected because they received the three highest criterion weights in the baseline AHP analysis and therefore had the greatest potential influence on the prioritization results. Each criterion was removed from the model individually, and the weights of the remaining criteria were re-normalized to sum to one. The AHP-weighted scoring results were then recalculated, and the resulting medical device rankings were compared with the baseline AHP-weighted scoring ranking to assess the effect of removing each criterion.

### 2.9. Comparative Evaluation Framework

To evaluate the robustness of the proposed repair prioritization framework, two comparative scenarios were developed based on pairwise comparison judgments provided by HE. These scenarios represent individual expert judgment and the hospital’s criticality-class logic, respectively. The resulting rankings were compared with those obtained from the proposed framework.

#### 2.9.1. Scenario 1: Case Hospital Expert-Based AHP

In this scenario, the AHP pairwise comparison matrix was constructed based on the individual judgments of HE. The evaluations were made independently of the hospital’s existing device criticality classes and current repair prioritization practice, while retaining the same evaluation criteria used in the proposed framework. This scenario was designed to provide a direct comparison between the proposed consensus-based framework and the individual prioritization perspective of an experienced biomedical professional working at the case hospital.

#### 2.9.2. Scenario 2: Class-Based AHP

For the class-based AHP scenario, the medical devices were first grouped according to the hospital’s existing criticality classification as Class 1, Class 2, and Class 3. Separate AHP pairwise comparison matrices were constructed for each criticality class based on the hospital expert’s judgments, resulting in class-specific criterion weights. The devices within each class were then evaluated and ranked using the corresponding class-specific AHP weights. The final class-based AHP ranking preserved the hospital’s class hierarchy, with Class 1 devices ranked first, followed by Class 2 and Class 3 devices, while the ordering of devices within each class was determined by their respective AHP-weighted scores.

### 2.10. Comparative Ranking Analysis

A comparative ranking analysis was conducted to evaluate the similarities and differences among the medical device priority rankings obtained using different decision-making approaches. In this analysis, the level of agreement between the ranking results was assessed using Spearman’s rank correlation coefficient, while the extent and magnitude of ranking variations were evaluated using NRC and MRS.

First, Spearman’s rank correlation coefficient was calculated to determine the degree of agreement between the rankings obtained from different prioritization methods. Spearman’s rank correlation is a non-parametric statistical measure that evaluates the strength and direction of the association between two ranked variables. A coefficient approaching ρ = 1 indicates a strong positive correlation between the rankings, ρ = 0 indicates no correlation, and a coefficient approaching ρ = −1 indicates a strong negative correlation. Spearman’s rank correlation coefficient was calculated using Equation (21) [32].

(21) $$\rho =1-\frac{6\sum_{i=1}^{n}d_{i}^{2}}{n(n^{2}-1)}$$

In Equation (21), $d_{i}$ represents the difference between the ranking positions of the same medical device obtained from two different prioritization methods, and n denotes the total number of medical devices.

To quantify ranking variations, the priority shift (PS) of each medical device was calculated as the difference between its ranking positions obtained from two different prioritization methods, as expressed in Equation (22).

(22) $$PS_{i}=R_{iB}-R_{iA}$$

In Equation (22), $PS_{i}$ denotes the priority shift of the ith medical device, while $R_{iA}$ and $R_{iB}$ represent its ranking positions under methods A and B, respectively. A value of $PS_{i}<0$ indicates that the medical device received a higher priority under the second method, $PS_{i}>0$ indicates a lower priority, and $PS_{i}=0$ indicates that its ranking position remained unchanged.

Based on the calculated priority shift values, two summary indicators were derived. NRC represents the total number of medical devices whose ranking positions differ between two prioritization methods; that is, a medical device is considered to have changed its ranking when $PS_{i}\neq 0$. MRS represents the largest absolute priority shift observed among all medical devices and is calculated as the maximum value of |$PS_{i}$|. These indicators provide a concise measure of the extent and magnitude of ranking variations between alternative prioritization approaches.

## 3. Results

This section presents the results obtained from the proposed medical device repair prioritization framework. First, the criterion weights and repair priority rankings obtained using the AHP-weighted scoring and TOPSIS approaches are presented, followed by the Fuzzy AHP criterion weights and Fuzzy TOPSIS ranking results. The robustness of the proposed framework is then evaluated through AHP- and TOPSIS-based sensitivity analyses under ±10%, ±30%, and ±50% criterion-weight variations, followed by criterion-removal analyses. Subsequently, the results of the hospital expert-based AHP and class-based AHP scenarios are presented. Finally, the rankings obtained from the main and comparative approaches are evaluated using Spearman’s rank correlation coefficient, NRC, and MRS to assess their agreement and ranking variations.

### 3.1. AHP Results

Table 8 presents the pairwise comparison matrix used to determine the relative importance of the evaluation criteria, together with the resulting AHP criterion weights. The calculated consistency ratio (CR = 0.001558) was well below the acceptable threshold of 0.10, indicating a high level of consistency in the pairwise comparisons. The evaluation scores assigned to the 32 medical devices for the seven criteria are presented in Table 9 and were subsequently used in both the AHP-weighted scoring and TOPSIS analyses. Based on the derived criterion weights and device evaluation scores, the repair priority results obtained using the AHP-weighted scoring approach are presented in Table 10.

### 3.2. TOPSIS Results

Table 11 presents the medical device repair prioritization results obtained using the TOPSIS method. The devices were ranked in descending order according to their CC values, with higher CC values indicating higher repair priority.

### 3.3. Fuzzy AHP Criterion Weights and Fuzzy TOPSIS Results

The Fuzzy AHP criterion-weighting and Fuzzy TOPSIS prioritization results are presented in Table 12 and Table 13, respectively. Table 12 presents the fuzzy criterion weights and the corresponding normalized crisp weights obtained using Fuzzy AHP. The fuzzy criterion weights were subsequently incorporated into the Fuzzy TOPSIS analysis to determine the repair priorities of the medical devices. Table 13 presents the resulting CC values and repair priority rankings, where higher CC values indicate higher repair priority.

### 3.4. Sensitivity Analysis Results

Table 14 and Table 15 present the sensitivity analysis results of the AHP-weighted scoring and TOPSIS models, respectively, under ±10%, ±30%, and ±50% variations in individual criterion weights. For each variation level, the resulting rankings were compared with their respective baseline rankings using Spearman’s rank correlation coefficient, NRC, and MRS. The results provide a comparative assessment of the robustness and ranking stability of both methods under progressively larger perturbations in criterion weights.

### 3.5. Targeted Criterion-Removal Robustness Analysis

Table 16 presents the results of the targeted criterion-removal robustness analysis, in which PSR, DF, and DC were individually excluded from the model. The resulting rankings were compared with the baseline AHP-weighted scoring ranking using Spearman’s rank correlation coefficient, NRC, and MRS.

### 3.6. Comparative Ranking Analysis Results

Table 17 and Table 18 present the repair priority rankings obtained from the hospital expert-based AHP and class-based AHP scenarios, respectively. In the hospital expert-based AHP scenario, criterion weights were derived from the pairwise comparison judgments provided by the case hospital expert, and the medical devices were ranked using the common 32 × 7 decision matrix employed in the main model. In the class-based AHP scenario, separate pairwise comparison matrices provided by the case hospital expert were used to represent the hospital’s criticality-class logic for Class 1, Class 2, and Class 3 devices. The device evaluation scores remained identical to those used in the main model. The pairwise comparison matrices underlying these comparative scenarios are provided in the Supplementary Materials (Tables S2–S5).

In the class-based AHP scenario, AHP-weighted scores were calculated separately within each device class and therefore represent relative repair priorities only among devices belonging to the same class. For the overall ranking, the hospital’s class hierarchy was preserved, with Class 1 devices prioritized over Class 2 devices and Class 2 devices prioritized over Class 3 devices.

Table 19 presents the comparative ranking analysis results across the alternative medical device repair prioritization approaches. The level of agreement between the rankings was evaluated using Spearman’s rank correlation coefficient, while NRC and MRS were used to quantify the extent and magnitude of ranking changes, respectively. All alternative rankings were compared with the baseline AHP-weighted scoring ranking. The medical devices exhibiting the most notable ranking changes in these comparisons are further examined in the following results and Section 4.

## 4. Discussion

The AHP criterion weights presented in Table 8 indicate a clear predominance of criteria with direct clinical relevance in determining repair priority. PSR received the highest weight (0.2619), followed by DF and DC, each with a weight of 0.2352. Together, PSR, DF, and DC account for approximately 73.24% of the total criterion weight. This finding is broadly consistent with previous medical equipment maintenance studies emphasizing risk, device function, and clinical criticality as central dimensions of maintenance prioritization. Taghipour et al. [17] incorporated function, mission criticality, and risk among the six main criteria of their hierarchical framework for medical equipment criticality assessment, whereas Jamshidi et al. [18] extended risk-based assessment by incorporating dimensions such as function and usage-related hazards. Similarly, Saleh et al. [19] considered function and physical risk within risk-based criteria and area criticality and device criticality within mission-based criteria. A more direct comparison can be made with the AHP results reported by Hutagalung and Hasibuan [23]. In their study, Risk received the highest weight of 0.340, followed by Class (0.214), Age (0.121), Degree of Importance of Mission (0.099), Recall and User Errors (0.095), Function (0.075), and Degree of Complexity of the Maintenance (0.057). Thus, the two studies show a clear convergence regarding the high importance assigned to patient safety/risk-related considerations. Nevertheless, the substantially greater importance assigned to DF (0.2352) in the present study is noteworthy. This difference may reflect not only differences in criterion structures but also the distinct decision contexts: rather than prioritizing a general maintenance program, the present study specifically addresses corrective repair priority after device failure has occurred. Although UF (0.0784) and AAD (0.0754) received substantially lower weights, their relative ordering is notably consistent with Hutagalung and Hasibuan [23]. Within their Degree of Importance of Mission dimension, utilization rate received a priority value of 0.542, compared with 0.458 for availability of alternative devices, indicating greater importance of utilization. The slightly higher weight of UF than AAD in the present study therefore follows the same relative direction. Moreover, utilization rate and availability of alternative devices were incorporated as sub-criteria by Taghipour et al. [17] and as additional dimensions of risk assessment by Jamshidi et al. [18]. The weight assigned to RSI (0.0754) indicates that resource- and maintenance-burden considerations remain relevant, although they are less influential than the clinically oriented criteria. While previous studies did not necessarily employ an integrated criterion identical to RSI, related dimensions have been considered in different forms. Taghipour et al. [17] included maintenance requirements as a main criterion, Saleh et al. [19] incorporated factors such as maintenance requirements, maintenance complexity, and downtime, and Zamzam et al. [20] employed a broader set of operational attributes including maintenance requirement, maintenance complexity, repair time, and downtime. Accordingly, RSI in the present framework consolidates several maintenance- and resource-related consequences that have often been represented separately in previous research into a broader dimension tailored to the corrective repair context. DA received the lowest criterion weight in Table 8 (0.0384). Age has nevertheless been incorporated into previous maintenance assessment frameworks, including those of Taghipour et al. [17], Jamshidi et al. [18], Zamzam et al. [20], and Hutagalung and Hasibuan [23]. Notably, Hutagalung and Hasibuan [23] assigned Age a weight of 0.121, exceeding both Function (0.075) and Degree of Complexity of the Maintenance (0.057), whereas the present study exhibits the opposite pattern, with DF (0.2352) substantially outweighing DA (0.0384). This distinction highlights an important characteristic of the corrective repair context: although device age may influence maintenance requirements, once a failure has occurred, the decision regarding which device should be repaired first appears to be driven more directly by its clinical function, potential patient-safety consequences, and clinical criticality. Overall, the weighting structure reported in Table 8 is largely consistent with the risk, function, criticality, utilization, alternative availability, maintenance-related, and age dimensions identified in previous studies, while revealing a distinct pattern in their relative importance within a corrective repair prioritization problem. In particular, the concentration of approximately 73.24% of the total weight in PSR, DF, and DC indicates that repair priority in the proposed framework is primarily driven by clinical consequences and continuity of healthcare delivery, whereas UF, AAD, RSI, and particularly DA serve as complementary discriminating factors.

The device-level results in Table 10 reflect the criterion-weight structure reported in Table 8, with devices associated with critical care, life support, and essential clinical functions generally concentrated near the top of the ranking. Anesthesia Machine (4.9233), External Cardiac Pacemaker (4.8862), Reusable ECG Patient Cables/Leadwires (4.8108), Electrocardiography (ECG) System (4.6997), and Mechanical Ventilator (4.6970) constitute the top five devices. This pattern is consistent with the combined weight of approximately 73.24% assigned to PSR, DF, and DC, indicating that patient-safety consequences, medical function, and clinical criticality are dominant determinants of corrective repair priority. A particularly noteworthy finding is the high ranking of Reusable ECG Patient Cables/Leadwires and Electrocardiography (ECG) System, which rank third and fourth despite being classified as Class 3 and Class 2, respectively. Conversely, Infusion Pump (for critical medications), although classified as Class 1, ranks only 16th with an AHP Score of 3.3906. These results demonstrate that the proposed framework does not simply reproduce predefined device classes. Rather, corrective repair priority emerges from the integrated seven-criterion profile, allowing devices with different conventional criticality classifications to move upward or downward according to their clinical, functional, and operational characteristics. The results are also generally consistent with the common devices evaluated by Taghipour et al. [17]. External Cardiac Pacemaker had a relatively high device risk value of 0.8542 in [17] and ranks second (4.8862) in the present study. Similarly, External Defibrillator and Intra-Aortic Balloon Pump (IABP) had device risk values of 0.7903 and 0.7845, respectively, in [17] and rank sixth (4.6970) and eighth (4.6203) in the present study. These parallels indicate that devices associated with substantial clinical consequences also tend to receive relatively high priority under the proposed framework. However, the device risk values in [17] were derived from specific failure modes and should not be interpreted as directly equivalent to the weighted AHP Scores obtained here. The methodological distinction is particularly evident for Mechanical Ventilator. Although its device risk value in [17] was 0.3911, it ranks fifth in the present study with an AHP Score of 4.6970. This difference reflects the specific objective of the proposed framework: determining corrective repair priority after device failure through the combined consideration of PSR, DF, DC, UF, AAD, RSI, and DA, rather than evaluating failure-mode risk alone. The intermediate positions of advanced diagnostic devices further illustrate the multi-criteria nature of the ranking. Computed Tomography (CT) Scanner and Magnetic Resonance Imaging (MRI) Scanner rank 12th and 13th, respectively, with identical AHP Scores of 3.8242. Although both receive high scores for DF, DC, UF, and RSI, their PSR score of 3 and AAD score of 1 moderate their overall repair priority. At the lower end, Water Bath (1.0754), Treadmill (1.5472), Hematology Staining Device (Stainer) (1.5489), and Examination Lamp (1.8472) receive substantially lower priorities. Taken together, these findings show that the resulting ranking neither simply follows conventional device classes nor reproduces failure-risk rankings from previous studies. Instead, it provides a corrective repair-specific prioritization structure in which clinical risk, device function, criticality, utilization, substitutability, resource impact, and age are evaluated jointly.

The comparative analysis in Table 19 indicates strong overall robustness of the Baseline AHP-Weighted Scoring across alternative prioritization approaches. Agreement was particularly high with Fuzzy TOPSIS (ρ = 0.9952), hospital expert-based AHP (ρ = 0.9908), and TOPSIS (ρ = 0.9901), indicating that the overall prioritization structure was largely preserved across different methodological and expert-based evaluations. In contrast, class-based AHP showed a comparatively lower, although still strong, correlation (ρ = 0.8328; NRC = 25; MRS = 21). This greater divergence is particularly informative because the class-based approach represents prioritization primarily through predefined device classes, whereas the baseline framework integrates clinical, functional, operational, and resource-related dimensions across seven criteria. Thus, the lower agreement with class-based AHP suggests that the proposed framework does not merely reproduce conventional device classifications but provides additional discrimination based on the multidimensional characteristics relevant to corrective repair priority.

The sensitivity analyses presented in Table 14 and Table 15 demonstrate a high degree of robustness of both the AHP-Weighted Scoring and TOPSIS rankings to changes in criterion weights. For the AHP-Weighted Scoring model, ±10% changes produced only limited rank variations, with Spearman correlations ranging from 0.9985 to 1.0000, a maximum rank shift of only two positions, and several scenarios producing no rank changes. Even under the substantially larger ±30% and ±50% perturbations, the overall ranking structure remained highly stable. At ±50%, Spearman correlations remained between 0.9901 and 0.9993, despite some localized changes among closely ranked devices. The greatest change occurred when PSR was increased by 50%, where Electrocardiography (ECG) System shifted from fourth to tenth position (MRS = 6). AAD and RSI also generated comparatively greater changes under large perturbations; for example, decreasing AAD by 50% produced 15 rank changes and an MRS of 4. Nevertheless, these changes did not result in a general restructuring of the ranking, as indicated by the consistently high rank correlations. TOPSIS showed a similarly robust, although slightly more responsive, pattern. Under ±10% changes, Spearman correlations ranged from 0.9963 to 1.0000, whereas under ±50% changes they remained between 0.9886 and 0.9993. The largest response occurred when PSR was reduced by 50%, producing 18 rank changes, an MRS of 5, and a shift of Electrocardiography (ECG) System from eighth to third position. Decreasing AAD by 50% also affected 17 devices, although the maximum displacement remained limited to three positions. Importantly, even these deliberately large ±50% perturbations preserved very high overall rank correlations. Thus, the sensitivity results indicate that the rankings are not dependent on small variations in the elicited criterion weights, while also identifying PSR and AAD as influential criteria under more extreme weight changes. Comparison of Table 14 and Table 15 further indicates that the AHP-Weighted Scoring ranking is somewhat more stable than the TOPSIS ranking, particularly in terms of the number of devices changing position. This difference is methodologically plausible because TOPSIS rankings depend not only on criterion weights but also on each alternative’s relative distance from the positive and negative ideal solutions; consequently, weight changes can modify the relative geometry among alternatives. In contrast, the AHP-Weighted Scoring model responds more directly to changes in the weighted criterion contributions. Nevertheless, the consistently high Spearman correlations obtained for both methods, including under ±50% perturbations, provide strong evidence that the principal prioritization structure is robust rather than being an artifact of a narrowly specified set of criterion weights.

The criterion-removal analysis in Table 16 was conducted to examine whether the conceptual relatedness of PSR, DF, and DC introduced substantial redundancy into the prioritization framework. Overall, the ranking remained highly stable after the individual exclusion of each criterion, with Spearman correlations of 0.9901, 0.9963, and 0.9905 following the removal of PSR, DF, and DC, respectively. Nevertheless, the resulting local rank changes demonstrate that the three criteria are not functionally interchangeable. Excluding PSR produced the largest number of rank changes (NRC = 18), with Electrocardiography (ECG) System moving from 4th to 1st and Infusion Pump (for critical medications) from 16th to 19th. Removal of DF had the smallest overall effect (ρ = 0.9963; NRC = 11; MRS = 2), whereas removal of DC also affected 11 devices but generated a larger maximum displacement (MRS = 4), most notably for Ultrasonic Cleaning Bath (22→26) and Patient Scale (26→22). These results support the conceptual distinction among the three criteria. PSR captures the potential patient-level consequence of device failure, DF represents the intrinsic medical function of the device, and DC reflects its context-dependent indispensability within clinical operations. If these criteria were largely redundant, removing any one of them would be expected to produce nearly identical effects on the ranking. Instead, the different devices affected and the different directions and magnitudes of rank changes indicate that each criterion contributes distinct information. At the same time, the consistently high Spearman correlations show that no single criterion independently determines the overall ranking. Thus, the criterion-removal analysis provides evidence that the proposed framework is both robust and non-redundant, while retaining clinically distinct dimensions of corrective repair priority.

Despite these contributions, several limitations should be acknowledged. First, the proposed framework was developed and evaluated using device data and expert assessments from a single hospital. Consequently, the criterion weights, device scores, and resulting repair priorities reflect the clinical, operational, and resource conditions of the participating institution. Hospitals may differ in their device inventories, clinical specialties, utilization patterns, availability of alternative devices, maintenance resources, and organizational priorities. Therefore, the resulting device ranking should not be interpreted as a universal repair-priority order applicable to all healthcare institutions. However, in terms of the scope of generalizability, this limitation primarily concerns the generalizability of the derived criterion weights, device scores, and resulting ranking rather than the applicability of the decision-support framework itself. The applicability of the framework across broader institutional contexts is supported by the fact that the selected criteria represent clinical, operational, and resource-related dimensions also identified in previous medical device maintenance studies and that the expert assessments were conducted by experienced professionals with knowledge of hospital and biomedical maintenance processes. Therefore, the specific device ranking obtained in the case hospital should not be generalized to other institutions; however, the proposed framework provides a structured approach that can be transferred to other hospital settings by reassessing the criterion weights and device scores according to local clinical, operational, and resource conditions. Future studies should validate the framework through multi-center applications involving hospitals with different characteristics and operational environments. Second, although the criteria were systematically defined and the final criterion set was established through expert evaluation, both the pairwise comparisons used to derive the AHP weights and the five-point device assessments involve expert judgment. Such judgments may vary according to professional experience, institutional responsibilities, and local operating conditions. The sensitivity analyses, alternative-method comparisons, and criterion-removal analysis performed in this study demonstrated a high degree of ranking robustness; nevertheless, these analyses do not eliminate the judgment-dependent nature of the input data. Future applications could involve larger and more diverse expert panels and, where available, complement expert assessments with objective operational data. Third, the proposed framework provides a static assessment of corrective repair priority based on the conditions represented at the time of evaluation. In actual hospital environments, some determinants of repair urgency may change over time. In particular, utilization intensity, availability of alternative devices, clinical demand, maintenance workload, and resource availability may vary dynamically. Accordingly, the priorities generated by the model should be periodically reassessed rather than regarded as permanently fixed. Future research could extend the framework into a dynamic decision-support system in which relevant criterion scores are updated as hospital conditions change. Finally, transferring the framework to other healthcare institutions may involve practical implementation challenges. Each institution would need to collect and standardize the information required for the seven criteria, establish an appropriate expert-evaluation process, and map its device inventory to the proposed scoring structure. Differences in data availability, recording practices, device nomenclature, and maintenance-management procedures may require institution-specific adaptations. Integration with existing computerized maintenance management systems (CMMS) or hospital asset-management systems could facilitate data collection and periodic updating of priorities; however, such integration may require data mapping, system configuration, and organizational coordination. Therefore, the proposed framework is intended as a transferable prioritization methodology rather than a fixed universal device ranking. Its criteria and analytical procedure can provide a common decision structure, while criterion weights and device scores should be reassessed according to the clinical and operational context of each institution. The broader applicability of AHP-based decision support in healthcare is also reflected in its use in other healthcare management contexts, such as the SWOT-AHP approach applied by Arian et al. [33] to address physician shortages.

Future research should first validate the proposed framework through multi-center applications involving hospitals with different clinical, operational, and resource characteristics. Such studies would enable the assessment of the transferability of the framework and the extent to which criterion weights and device priorities vary across institutional settings. Future extensions may also complement expert-based evaluations with objective and continuously updated operational data, including device utilization, maintenance history, downtime, repair time, and real-time equipment status. Integration with computerized maintenance management systems (CMMS) could facilitate this process and enable repair priorities to be updated dynamically as hospital conditions change. Furthermore, artificial intelligence and machine-learning approaches could be incorporated to exploit historical maintenance and failure data, identify evolving operational patterns, and support predictive information alongside the proposed corrective repair prioritization framework. These developments could ultimately transform the proposed approach from a periodic prioritization model into a more dynamic and adaptive decision-support system for medical device maintenance management.

## 5. Conclusions

This study developed a multi-criteria decision-support framework for prioritizing corrective repair interventions for medical devices after failure. The proposed approach integrates PSR, DF, DC, UF, AAD, RSI, and DA and combines AHP-derived criterion weights with device-level scoring. The results showed that PSR, DF, and DC were the dominant criteria, together accounting for approximately 73.24% of the total weight, while the resulting device priorities did not simply reproduce predefined device classes. Method comparisons, sensitivity analyses, and criterion-removal analyses further demonstrated that the overall prioritization structure remained highly robust under alternative methodological assumptions and changes in criterion importance. The main contribution of the study to health technology management is the transformation of corrective repair prioritization into a transparent, reproducible, and multidimensional decision-support process. The framework can support clinical engineering and maintenance units in determining which failed devices should receive earlier intervention when multiple repair demands compete for limited resources. However, implementation in other healthcare institutions requires local adaptation, including standardized data collection for the criteria, organization of the expert-evaluation process, and alignment with existing maintenance management systems. Accordingly, the framework should be considered a transferable methodology rather than a universally applicable device ranking, with criterion weights and device scores recalibrated according to institutional conditions. Future research should extend the proposed framework through multi-hospital validation and the integration of real-time operational data and machine-learning-based approaches, thereby supporting the development of more dynamic and adaptive medical device repair prioritization systems.

## Figures and Tables

**Table 1:** Evaluation criteria used for medical device repair prioritization.

Criterion	Description
PSR	Potential level of harm and risk that device failure may pose to patient safety and clinical outcomes.
DF	The primary medical function of the device in healthcare delivery, including its intrinsic and relatively stable role as supportive, analytical, diagnostic, therapeutic, or life-support equipment.
DC	The importance and indispensability of the device within current clinical care processes, reflecting its context-dependent criticality under prevailing clinical and operational conditions.
UF	The frequency and intensity with which the device is used in routine healthcare operations.
AAD	The availability and accessibility of backup or alternative devices capable of performing the same clinical function in the event of device failure.
RSI	The extent of operational disruption and resource pressure caused by device failure through its effects on workforce, clinical and maintenance scheduling, technical resources, and budget.
DA	The age-related maintenance requirement level, reflecting the increased likelihood of complex failures, diagnostic/technical assessment requirements, and the potential need for external technical support as the device ages.

**Table 2:** Scoring scale used for criteria evaluation.

Criterion	1	2	3	4	5
PSR	Negligible	Minor impact	Moderate risk	Serious risk	Life-threatening risk
DF	Supportive	Analytical	Diagnostic	Therapeutic	Life-support
DC	Low	Limited	Moderate	High	Critical
UF	Rarely used	Low	Moderate	High	Continuous use
AAD	Multiple alternatives available	Easily accessible alternative	Limited alternatives	Difficult to obtain alternative	No alternative available
RSI	Low	Limited	Moderate	High	Very high
DA	<3 years	3–5 years	5–7 years	7–10 years	>10 years

**Table 3:** Medical devices included in the analysis and their criticality classes.

Class	Medical Device
1	Intra-Aortic Balloon Pump (IABP)
1	Mechanical Ventilator
1	External Defibrillator
1	Neonatal Incubator
1	External Cardiac Pacemaker
1	Anesthesia Machine
1	Dialysis/Hemofiltration Unit
1	Cardiopulmonary Bypass Device (CPB)
1	Extracorporeal Membrane Oxygenation (ECMO) System
1	Infusion Pump (for critical medications)
2	Computed Tomography (CT) Scanner
2	Magnetic Resonance Imaging (MRI) Scanner
2	Gamma Camera
2	Doppler Ultrasound System
2	Electrocardiography (ECG) System
2	Fetal Monitor
2	Laboratory Autoanalyzer
2	Intensive Care Monitoring System (Telemetry Monitor)
2	Phototherapy Unit
2	Echocardiography (ECHO) System
2	General Patient Monitor
2	X-ray System
2	Sterilization Unit (Autoclave)
3	Examination Lamp
3	Patient Scale
3	Treadmill
3	Hematology Staining Device (Stainer)
3	Ultrasonic Cleaning Bath
3	Water Bath
3	Reusable ECG Patient Cables/Leadwires
3	Dental Equipment
3	Medical Sterilizer

**Table 4:** Professional backgrounds, experience, expertise, and roles of the experts.

Expert	Professional Background	Professional Experience	Relevant Experience/Expertise	Role in the Study
Expert 1 (E1)	Biomedical Engineer	4 years	Professional experience in biomedical devices, device maintenance, and related processes in hospital settings	Evaluation and approval of the suitability of the 32 medical devices proposed by the case-hospital expert for inclusion in the study; contribution to the identification of the evaluation criteria; participation in the joint evaluation of the 32 devices using the seven criteria and the 1–5 scoring scale; participation in establishing, through expert consensus, the AHP pairwise comparison matrix used to derive the criterion weights for the main model
Expert 2 (E2)	Biomedical Technician/Manager	5 years	Professional and managerial experience in biomedical device maintenance and management processes in hospital settings	Evaluation and approval of the suitability of the 32 medical devices proposed by the case-hospital expert for inclusion in the study; contribution to the identification of the evaluation criteria; participation in the joint evaluation of the 32 devices using the seven criteria and the 1–5 scoring scale; participation in establishing, through expert consensus, the AHP pairwise comparison matrix used to derive the criterion weights for the main model
Expert 3 (E3)	Engineer/Co-founder of a company operating in the medical device sector	3–4 years	Experience in medical device maintenance, particularly surgical instruments and sterilization systems; professional contracts with 27 hospitals and active consultancy services to 5 hospitals through his company	Evaluation and approval of the suitability of the 32 medical devices proposed by the case-hospital expert for inclusion in the study; contribution to the identification of the evaluation criteria; participation in the joint evaluation of the 32 devices using the seven criteria and the 1–5 scoring scale; participation in establishing, through expert consensus, the AHP pairwise comparison matrix used to derive the criterion weights for the main model
Hospital Expert (HE)	Biomedical Device Technician	12 years	Extensive professional experience in biomedical device maintenance and repair processes in hospital settings; direct knowledge of the case hospital’s device inventory, maintenance processes, and existing repair-prioritization practices	Proposal of the 32 medical devices for inclusion in the study based on the hospital’s maintenance management system and operational conditions; contribution to the identification of the final evaluation criteria; participation in the joint evaluation of the 32 devices using the seven criteria and the 1–5 scoring scale; development of the pairwise comparison matrices used in the hospital expert-based AHP and class-based AHP scenarios

**Table 5:** Saaty’s pairwise comparison scale [27].

Value	Definition
1	Equal importance
3	Moderate importance
5	Strong importance
7	Very strong importance
9	Extreme importance
2, 4, 6, 8	Intermediate values
1/x	Reciprocal value

**Table 6:** RI values [27].

Number of Criteria	3	4	5	6	7	8	9	10
RI	0.58	0.90	1.12	1.24	1.32	1.41	1.45	1.49

**Table 7:** Conversion of Saaty’s scale into TFNs.

Linguistic Term	Saaty Scale	TFN Equivalent
Equal	1	(1, 1, 1)
Moderate	3	(2, 3, 4)
Strong	5	(4, 5, 6)
Very Strong	7	(6, 7, 8)
Extreme	9	(8, 9, 9)
Intermediate Values	2, 4, 6, 8	(1, 2, 3), (3, 4, 5), (5, 6, 7), (7, 8, 9)
$\mathrm{Reciprocal}\;\mathrm{Values}\;(1/a_{ij}$)	1/9…1/3	$\left(1/u_{ji},1/m_{ji},1/l_{ji}\right)$

**Table 8:** Pairwise comparison matrix and resulting AHP criterion weights.

Criterion	PSR	DF	DC	UF	AAD	RSI	DA	AHP Weight
PSR	1.000	1.000	1.000	3.000	4.000	4.000	7.000	0.261903
DF	1.000	1.000	1.000	3.000	3.000	3.000	6.000	0.235244
DC	1.000	1.000	1.000	3.000	3.000	3.000	6.000	0.235244
UF	0.333	0.333	0.333	1.000	1.000	1.000	2.000	0.078415
AAD	0.250	0.333	0.333	1.000	1.000	1.000	2.000	0.075421
RSI	0.250	0.333	0.333	1.000	1.000	1.000	2.000	0.075421
DA	0.143	0.167	0.167	0.500	0.500	0.500	1.000	0.038352

**Table 9:** Medical device evaluation scores used in the AHP-weighted scoring and TOPSIS analyses.

Class	Medical Device	PSR	DF	DC	UF	AAD	RSI	DA
1	Intra-Aortic Balloon Pump (IABP)	5	5	5	5	2	5	1
1	Mechanical Ventilator	5	5	5	5	4	3	3
1	External Defibrillator	5	5	5	5	4	3	3
1	Neonatal Incubator	5	5	5	5	2	3	3
1	External Cardiac Pacemaker	5	5	5	5	4	5	4
1	Anesthesia Machine	5	5	5	5	5	5	3
1	Dialysis/Hemofiltration Unit	5	5	5	5	4	3	2
1	Cardiopulmonary Bypass Device (CPB)	5	5	5	4	4	3	3
1	Extracorporeal Membrane Oxygenation (ECMO) System	5	5	5	3	3	4	1
1	Infusion Pump (for critical medications)	4	4	4	2	1	1	4
2	Computed Tomography (CT) Scanner	3	5	4	5	1	5	2
2	Magnetic Resonance Imaging (MRI) Scanner	3	5	4	5	1	5	2
2	Gamma Camera	3	5	4	3	1	5	1
2	Doppler Ultrasound System	3	4	3	4	3	3	2
2	Electrocardiography (ECG) System	4	5	5	5	5	5	4
2	Fetal Monitor	2	3	2	4	2	1	4
2	Laboratory Autoanalyzer	3	4	3	3	2	2	3
2	Intensive Care Monitoring System (Telemetry Monitor)	1	3	2	4	4	1	4
2	Phototherapy Unit	1	2	1	2	4	1	4
2	Echocardiography (ECHO) System	3	4	3	4	2	4	3
2	General Patient Monitor	1	2	1	1	5	1	5
2	X-ray System	3	3	2	3	2	4	3
2	Sterilization Unit (Autoclave)	2	4	2	2	1	1	3
3	Examination Lamp	1	2	1	3	5	1	5
3	Patient Scale	1	4	1	2	3	2	5
3	Treadmill	1	3	1	1	1	1	3
3	Hematology Staining Device (Stainer)	1	2	2	2	1	1	1
3	Ultrasonic Cleaning Bath	1	4	3	3	1	2	2
3	Water Bath	1	1	1	1	1	2	1
3	Reusable ECG Patient Cables/Leadwires	5	5	5	5	4	4	4
3	Dental Equipment	3	4	3	4	2	3	3
3	Medical Sterilizer	4	5	4	3	1	2	2

**Table 10:** Medical device repair prioritization results obtained using AHP-weighted scoring.

Priority Ranking	Class	Medical Device	AHP-Weighted Score
1	1	Anesthesia Machine	4.923296
2	1	External Cardiac Pacemaker	4.886227
3	3	Reusable ECG Patient Cables/Leadwires	4.810806
4	2	Electrocardiography (ECG) System	4.699745
5	1	Mechanical Ventilator	4.697034
6	1	External Defibrillator	4.697034
7	1	Dialysis/Hemofiltration Unit	4.658682
8	1	Intra-Aortic Balloon Pump (IABP)	4.620330
9	1	Cardiopulmonary Bypass Device (CPB)	4.618619
10	1	Neonatal Incubator	4.546192
11	1	Extracorporeal Membrane Oxygenation (ECMO) System	4.463500
12	2	Computed Tomography (CT) Scanner	3.824211
13	2	Magnetic Resonance Imaging (MRI) Scanner	3.824211
14	3	Medical Sterilizer	3.703022
15	2	Gamma Camera	3.629029
16	1	Infusion Pump (for critical medications)	3.390646
17	2	Echocardiography (ECHO) System	3.313659
18	2	Doppler Ultrasound System	3.275307
19	3	Dental Equipment	3.238238
20	2	Laboratory Autoanalyzer	3.084403
21	2	X-ray System	2.764756
22	3	Ultrasonic Cleaning Bath	2.446824
23	2	Fetal Monitor	2.393357
24	2	Sterilization Unit (Autoclave)	2.357999
25	2	Intensive Care Monitoring System (Telemetry Monitor)	2.282296
26	3	Patient Scale	2.163818
27	3	Examination Lamp	1.847165
28	2	General Patient Monitor	1.690335
29	2	Phototherapy Unit	1.654977
30	3	Hematology Staining Device (Stainer)	1.548904
31	3	Treadmill	1.547193
32	3	Water Bath	1.075421

**Table 11:** Medical device repair prioritization results obtained using TOPSIS.

Priority Ranking	Class	Medical Device	CC
1	1	Anesthesia Machine	0.951936
2	1	External Cardiac Pacemaker	0.945377
3	3	Reusable ECG Patient Cables/Leadwires	0.929131
4	1	Mechanical Ventilator	0.889935
5	1	External Defibrillator	0.889935
6	1	Cardiopulmonary Bypass Device (CPB)	0.883292
7	1	Dialysis/Hemofiltration Unit	0.879770
8	2	Electrocardiography (ECG) System	0.852834
9	1	Extracorporeal Membrane Oxygenation (ECMO) System	0.846500
10	1	Intra-Aortic Balloon Pump (IABP)	0.841364
11	1	Neonatal Incubator	0.836172
12	3	Medical Sterilizer	0.693196
13	2	Computed Tomography (CT) Scanner	0.645932
14	2	Magnetic Resonance Imaging (MRI) Scanner	0.645932
15	1	Infusion Pump (for critical medications)	0.645768
16	2	Gamma Camera	0.632920
17	2	Echocardiography (ECHO) System	0.556214
18	2	Doppler Ultrasound System	0.555861
19	3	Dental Equipment	0.547738
20	2	Laboratory Autoanalyzer	0.531885
21	2	X-ray System	0.433157
22	3	Ultrasonic Cleaning Bath	0.378619
23	2	Sterilization Unit (Autoclave)	0.369723
24	2	Fetal Monitor	0.330892
25	3	Patient Scale	0.309546
26	2	Intensive Care Monitoring System (Telemetry Monitor)	0.299968
27	3	Examination Lamp	0.228529
28	2	General Patient Monitor	0.217168
29	3	Treadmill	0.206799
30	2	Phototherapy Unit	0.187027
31	3	Hematology Staining Device (Stainer)	0.171329
32	3	Water Bath	0.046389

**Table 12:** Criterion weights obtained using Fuzzy AHP.

Criterion	Fuzzy Weight	Crisp Weight
PSR	(0.1573, 0.2614, 0.3402)	0.2342
DF	(0.1364, 0.2355, 0.3131)	0.2114
DC	(0.1364, 0.2355, 0.3131)	0.2114
UF	(0.0445, 0.0785, 0.1138)	0.0731
AAD	(0.0525, 0.0753, 0.1746)	0.0933
RSI	(0.0525, 0.0753, 0.1746)	0.0933
DA	(0.0358, 0.0384, 0.1957)	0.0833

**Table 13:** Medical device repair prioritization results obtained using Fuzzy TOPSIS.

Priority Ranking	Class	Medical Device	CC
1	1	External Cardiac Pacemaker	0.5725
2	1	Anesthesia Machine	0.5686
3	3	Reusable ECG Patient Cables/Leadwires	0.5684
4	2	Electrocardiography (ECG) System	0.5627
5	1	Mechanical Ventilator	0.5512
6	1	External Defibrillator	0.5512
7	1	Cardiopulmonary Bypass Device (CPB)	0.5471
8	1	Dialysis/Hemofiltration Unit	0.542
9	1	Neonatal Incubator	0.5317
10	1	Intra-Aortic Balloon Pump (IABP)	0.5265
11	1	Extracorporeal Membrane Oxygenation (ECMO) System	0.5212
12	2	Computed Tomography (CT) Scanner	0.4755
13	2	Magnetic Resonance Imaging (MRI) Scanner	0.4755
14	3	Medical Sterilizer	0.4643
15	2	Gamma Camera	0.4539
16	1	Infusion Pump (for critical medications)	0.4533
17	2	Echocardiography (ECHO) System	0.4531
18	2	Doppler Ultrasound System	0.4438
19	3	Dental Equipment	0.4432
20	2	Laboratory Autoanalyzer	0.4247
21	2	X-ray System	0.399
22	2	Fetal Monitor	0.3585
23	2	Intensive Care Monitoring System (Telemetry Monitor)	0.3562
24	3	Ultrasonic Cleaning Bath	0.3531
25	2	Sterilization Unit (Autoclave)	0.3446
26	3	Patient Scale	0.3425
27	3	Examination Lamp	0.3058
28	2	Phototherapy Unit	0.2923
29	2	General Patient Monitor	0.2889
30	3	Treadmill	0.2639
31	3	Hematology Staining Device (Stainer)	0.2464
32	3	Water Bath	0.2011

**Table 14:** AHP-weighted scoring sensitivity analysis results under ±10%, ±30%, and ±50% criterion weight variations.

Sensitivity Scenario	ρ	NRC	MRS
PSR (+10%)	0.9989	3	2
PSR (−10%)	1	0	0
DF (+10%)	0.9996	2	1
DF (−10%)	1	0	0
DC (+10%)	1	0	0
DC (−10%)	0.9996	2	1
UF (+10%)	1	0	0
UF (−10%)	0.9993	4	1
AAD (+10%)	0.9996	2	1
AAD (−10%)	0.9989	3	2
RSI (+10%)	1	0	0
RSI (−10%)	0.9985	5	2
DA (+10%)	0.9993	4	1
DA (−10%)	0.9989	3	2
PSR (+30%)	0.9934	9	5
PSR (−30%)	0.9989	6	1
DF (+30%)	0.9993	4	1
DF (−30%)	0.9993	4	1
DC (+30%)	1	0	0
DC (−30%)	0.9989	6	1
UF (+30%)	1	0	0
UF (−30%)	0.9989	6	1
AAD (+30%)	0.9996	2	1
AAD (−30%)	0.9985	5	2
RSI (+30%)	0.9993	4	1
RSI (−30%)	0.9974	6	3
DA (+30%)	0.9993	4	1
DA (−30%)	0.9989	3	2
PSR (+50%)	0.9901	13	6
PSR (−50%)	0.9971	10	2
DF (+50%)	0.9971	8	3
DF (−50%)	0.9993	4	1
DC (+50%)	0.9989	3	2
DC (−50%)	0.9971	10	2
UF (+50%)	0.9989	6	1
UF (−50%)	0.9982	7	2
AAD (+50%)	0.9989	6	1
AAD (−50%)	0.9905	15	4
RSI (+50%)	0.9971	8	3
RSI (−50%)	0.9963	9	3
DA (+50%)	0.9993	4	1
DA (−50%)	0.9989	3	2

**Table 15:** TOPSIS sensitivity analysis results under ±10%, ±30%, and ±50% criterion weight variations.

Sensitivity Scenario	ρ	NRC	MRS
PSR (+10%)	0.9963	9	3
PSR (−10%)	0.9996	2	1
DF (+10%)	0.9996	2	1
DF (−10%)	0.9985	5	2
DC (+10%)	0.9989	3	2
DC (−10%)	0.9996	2	1
UF (+10%)	1	0	0
UF (−10%)	0.9989	3	2
AAD (+10%)	0.9996	2	1
AAD (−10%)	0.9982	7	2
RSI (+10%)	1	0	0
RSI (−10%)	0.9985	5	2
DA (+10%)	0.9989	3	2
DA (−10%)	0.9996	2	1
PSR (+30%)	0.9963	9	3
PSR (−30%)	0.9941	14	4
DF (+30%)	0.9978	9	2
DF (−30%)	0.9974	8	2
DC (+30%)	0.9971	10	2
DC (−30%)	0.9996	2	1
UF (+30%)	0.9982	7	2
UF (−30%)	0.9985	5	2
AAD (+30%)	0.9989	6	1
AAD (−30%)	0.9938	14	3
RSI (+30%)	0.9996	2	1
RSI (−30%)	0.9982	7	2
DA (+30%)	0.9974	8	2
DA (−30%)	0.9989	6	1
PSR (+50%)	0.9956	10	3
PSR (−50%)	0.9886	18	5
DF (+50%)	0.9963	11	2
DF (−50%)	0.9952	12	3
DC (+50%)	0.9945	12	4
DC (−50%)	0.9993	4	1
UF (+50%)	0.9982	7	2
UF (−50%)	0.9985	5	2
AAD (+50%)	0.9989	6	1
AAD (−50%)	0.9912	17	3
RSI (+50%)	0.9956	9	4
RSI (−50%)	0.9974	8	2
DA (+50%)	0.9974	8	2
DA (−50%)	0.9982	7	2

**Table 16:** Results of the targeted criterion-removal robustness analysis.

Criterion-Removal Scenario	ρ	NRC	MRS
PSR excluded	0.9901	18	3
DF excluded	0.9963	11	2
DC excluded	0.9905	11	4

**Table 17:** Hospital expert-based AHP repair prioritization results.

Priority Ranking	Class	Medical Device	AHP-Weighted Score
1	1	Anesthesia Machine	4.927301
2	1	External Cardiac Pacemaker	4.900257
3	3	Reusable ECG Patient Cables/Leadwires	4.845868
4	1	Mechanical Ventilator	4.755130
5	1	External Defibrillator	4.755130
6	1	Dialysis/Hemofiltration Unit	4.718781
7	1	Intra-Aortic Balloon Pump (IABP)	4.664421
8	1	Cardiopulmonary Bypass Device (CPB)	4.634210
9	1	Neonatal Incubator	4.628343
10	2	Electrocardiography (ECG) System	4.584149
11	1	Extracorporeal Membrane Oxygenation (ECMO) System	4.431586
12	2	Computed Tomography (CT) Scanner	3.728079
13	2	Magnetic Resonance Imaging (MRI) Scanner	3.728079
14	3	Medical Sterilizer	3.702575
15	2	Gamma Camera	3.449890
16	1	Infusion Pump (for critical medications)	3.404814
17	2	Echocardiography (ECHO) System	3.307067
18	2	Doppler Ultrasound System	3.279723
19	3	Dental Equipment	3.252679
20	2	Laboratory Autoanalyzer	3.077370
21	2	X-ray System	2.840700
22	2	Fetal Monitor	2.455303
23	2	Sterilization Unit (Autoclave)	2.308872
24	3	Ultrasonic Cleaning Bath	2.218624
25	2	Intensive Care Monitoring System (Telemetry Monitor)	2.202588
26	3	Patient Scale	2.032951
27	3	Examination Lamp	1.835965
28	2	Phototherapy Unit	1.615302
29	2	General Patient Monitor	1.594125
30	3	Hematology Staining Device (Stainer)	1.466367
31	3	Treadmill	1.463004
32	3	Water Bath	1.054389

**Table 18:** Class-based AHP repair prioritization results.

Priority Ranking	Class	Medical Device	AHP-Weighted Score
1	1	Anesthesia Machine	4.913057
2	1	External Cardiac Pacemaker	4.877673
3	1	Mechanical Ventilator	4.747259
4	1	External Defibrillator	4.747259
5	1	Dialysis/Hemofiltration Unit	4.703788
6	1	Cardiopulmonary Bypass Device (CPB)	4.608464
7	1	Intra-Aortic Balloon Pump (IABP)	4.589548
8	1	Neonatal Incubator	4.589548
9	1	Extracorporeal Membrane Oxygenation (ECMO) System	4.347343
10	1	Infusion Pump (for critical medications)	3.355430
11	2	Electrocardiography (ECG) System	4.744555
12	2	Computed Tomography (CT) Scanner	3.782427
13	2	Magnetic Resonance Imaging (MRI) Scanner	3.782427
14	2	Gamma Camera	3.504426
15	2	Echocardiography (ECHO) System	3.315873
16	2	Doppler Ultrasound System	3.263491
17	2	Laboratory Autoanalyzer	2.977444
18	2	X-ray System	2.796937
19	2	Intensive Care Monitoring System (Telemetry Monitor)	2.443192
20	2	Fetal Monitor	2.420636
21	2	Sterilization Unit (Autoclave)	2.232888
22	2	General Patient Monitor	1.863828
23	2	Phototherapy Unit	1.811447
24	3	Reusable ECG Patient Cables/Leadwires	4.735486
25	3	Medical Sterilizer	3.451626
26	3	Dental Equipment	3.207375
27	3	Ultrasonic Cleaning Bath	2.414750
28	3	Patient Scale	2.254023
29	3	Examination Lamp	2.075089
30	3	Hematology Staining Device (Stainer)	1.528111
31	3	Treadmill	1.508525
32	3	Water Bath	1.104266

**Table 19:** Comparative ranking analysis results across alternative repair prioritization approaches.

Compared Rankings	ρ	NRC	MRS
Baseline AHP-Weighted Scoring vs. TOPSIS	0.9901	19	4
Baseline AHP-Weighted Scoring vs. Fuzzy TOPSIS	0.9952	14	2
Baseline AHP-Weighted Scoring vs. Hospital Expert-Based AHP	0.9908	12	6
Baseline AHP-Weighted Scoring vs. Class-Based AHP	0.8328	25	21

## Data Availability

Data are contained within the article and Supplementary Materials.

## Supplementary Materials

The following supporting information can be downloaded at: https://www.mdpi.com/article/10.3390/healthcare14172799/s1. Table S1: Clinical and Operational Characteristics of the Medical Devices Included in the Analysis; Table S2: Pairwise Comparison Matrix Used in the Hospital Expert-Based AHP Scenario; Table S3: Pairwise Comparison Matrix Used for Class 1 Devices in the Class-Based AHP Scenario; Table S4: Pairwise Comparison Matrix Used for Class 2 Devices in the Class-Based AHP Scenario; Table S5: Pairwise Comparison Matrix Used for Class 3 Devices in the Class-Based AHP Scenario; Code S1: MATLAB Code for Fuzzy AHP Criterion Weight Calculation; Code S2: MATLAB Code for the Integrated Fuzzy AHP–Fuzzy TOPSIS Analysis.

## Abbreviations

The following abbreviations are used in this manuscript:

MCDM	Multi-Criteria Decision-Making
PSR	Patient Safety Risk
DF	Device Function
DC	Device Criticality
UF	Utilization Frequency
AAD	Availability of Alternative Devices
RSI	Resource Impact
DA	Device Age
AHP	Analytic Hierarchy Process
TOPSIS	Technique for Order Preference by Similarity to Ideal Solution
Fuzzy AHP	Fuzzy Analytic Hierarchy Process
Fuzzy TOPSIS	Fuzzy Technique for Order Preference by Similarity to Ideal Solution
MRI	Magnetic Resonance Imaging
NRC	Number of Rank Changes
MRS	Maximum Rank Shift
CR	Consistency Ratio
CI	Consistency Index
RI	Random Index
CC	Closeness Coefficient
PS	Priority Shift
TFN	Triangular Fuzzy Number
PIS	Positive Ideal Solution
NIS	Negative Ideal Solution
FPIS	Fuzzy Positive Ideal Solution
FNIS	Fuzzy Negative Ideal Solution
COA	Center of Area
E1	Expert 1
E2	Expert 2
E3	Expert 3
HE	Hospital Expert
