# Supplementary material for: A Multi-Criteria Decision Support Framework for Prioritizing the Repair of Failed Medical Devices: A Hospital Case Study
Source: Healthcare (Basel). 2026 Sep 1;14(17):2799. doi: 10.3390/healthcare14172799 (PMC13565927; doi:10.3390/healthcare14172799)
Supplement: Supplementary file 1 [file healthcare-14-02799-s001.zip › healthcare-4434830-supplementary.pdf]

Supplementary Materials

Table S1. Clinical and Operational Characteristics of the Medical Devices Included in the Analysis.

Medical Device	Primary Intended Use	Typical Clinical Setting/Unit	Significance of Failure
Intra-Aortic Balloon Pump (IABP)	Hemodynamic support	Cardiac intensive care, catheterization	Provides circulatory support for critically ill cardiac patients.
Mechanical Ventilator	Mechanical respiratory support	Intensive care, emergency, operating room	It is a life-support device; its failure may directly affect patient safety.
External Defibrillator	Defibrillation/cardioversion	Emergency, intensive care, operating room	Critical for intervention in cardiac arrest.
Neonatal Incubator	Providing a controlled thermal environment for neonates	Neonatal intensive care	May affect the safety of premature infants/neonates and continuity of care.
External Cardiac Pacemaker	Temporary cardiac pacing	Intensive care, emergency, cardiology	Provides direct life support in cases of critical bradycardia.
Anesthesia Machine	Providing anesthetic gases and ventilation	Operating room	May result in postponement of the surgical procedure or compromise patient ventilation.
Dialysis/Hemofiltration Unit	Renal replacement therapy	Dialysis, intensive care	Delayed treatment may have serious consequences for critically ill patients.
Cardiopulmonary Bypass Device (CPB)	Taking over heart-lung function during surgery	Cardiovascular surgery	Directly affects the performance of cardiac surgery.
Extracorporeal Membrane Oxygenation (ECMO) System	Extracorporeal cardiopulmonary support	Intensive care	It is an advanced life-support device.
Infusion Pump (for critical medications)	Controlled administration of medications/fluids	Intensive care, operating room	Important for administering critical medications at the correct rate.
Computed Tomography (CT) Scanner	Cross-sectional diagnostic imaging	Radiology, emergency	May cause delays in emergency diagnosis in cases such as trauma and stroke.
Magnetic Resonance Imaging (MRI) Scanner	Advanced diagnostic imaging	Radiology	May significantly reduce diagnostic service capacity.

Gamma Camera	Nuclear medicine imaging	Nuclear medicine	Causes disruption to diagnostic nuclear medicine services.
Doppler Ultrasound System	Blood flow/vascular assessment	Radiology, vascular, obstetrics	May affect vascular and obstetric assessments.
Electrocardiography (ECG) System	Recording of cardiac electrical activity	Cardiology, emergency	May cause delays in the diagnosis of acute cardiac conditions.
Fetal Monitor	Monitoring fetal heart rate and uterine activity	Obstetrics, delivery unit	May affect the early detection of fetal distress.
Laboratory Autoanalyzer	Automated laboratory analysis	Clinical laboratory	May cause delays in a large number of laboratory results.
Intensive Care Monitoring System (Telemetry Monitor)	Continuous/remote patient monitoring	Intensive care, cardiology	May affect the early detection of patient deterioration.
Phototherapy Unit	Treatment of neonatal hyperbilirubinemia	Neonatal care	May cause delays in treatment.
Echocardiography (ECHO) System	Cardiac ultrasonography	Cardiology	May delay cardiac diagnosis and treatment decisions.
General Patient Monitor	Monitoring of vital signs	Inpatient ward, emergency, intensive care	Affects monitoring of the patient's physiological condition.
X-ray System	Radiographic imaging	Radiology, emergency	May affect diagnostic processes and emergency services.
Sterilization Unit (Autoclave)	Steam sterilization	Central sterilization	Critical for the continuity of sterile surgical material availability.
Examination Lamp	Illumination of the examination area	Outpatient clinic, emergency	Affects workflow.
Patient Scale	Body weight measurement	Inpatient ward, outpatient clinic	May affect clinical decisions but generally has low acute criticality.
Treadmill	Exercise/stress testing	Cardiology	May cause disruption to elective cardiac assessments.
Hematology Staining Device (Stainer)	Staining of laboratory specimens	Hematology/pathology	May delay diagnostic laboratory workflow.
Ultrasonic Cleaning Bath	Ultrasonic pre-cleaning of instruments	Sterilization/biomedical/laboratory	May create bottlenecks in the reprocessing process and cause infection-control problems.

Water Bath	Temperature-controlled sample/process handling	Laboratory	May affect laboratory processes; its acute clinical impact is generally limited.
Reusable ECG Patient Cables/Leadwires	Transmission of the ECG signal from the patient to the system	Emergency, intensive care, cardiology	Causes disruption to ECG monitoring/diagnosis.
Dental Equipment	Dental diagnosis/treatment	Dental clinic	May cause interruption of the relevant service.
Medical Sterilizer	Sterilization process	Central sterilization	Affects sterile material production and continuity of operating-room services.

Table S2. Pairwise Comparison Matrix Used in the Hospital Expert-Based AHP Scenario.

Criterion	PSR	DF	DC	UF	AAD	RSI	DA
PSR	1.000	2.000	3.000	5.000	7.000	7.000	7.000
DF	0.500	1.000	2.000	3.000	3.000	3.000	3.000
DC	0.333	0.500	1.000	2.000	3.000	3.000	5.000
UF	0.200	0.333	0.500	1.000	2.000	5.000	5.000
AAD	0.143	0.333	0.333	0.500	1.000	2.000	2.000
RSI	0.143	0.333	0.333	0.200	0.500	1.000	3.000
DA	0.143	0.333	0.200	0.200	0.500	0.333	1.000

Table S3. Pairwise Comparison Matrix Used for Class 1 Devices in the Class-Based AHP Scenario.

Criterion (Class 1)	PSR	DF	DC	UF	AAD	RSI	DA
PSR	1.000	1.000	2.000	2.000	4.000	7.000	7.000
DF	1.000	1.000	2.000	2.000	3.000	6.000	6.000
DC	0.500	0.500	1.000	1.000	2.000	3.000	3.000
UF	0.500	0.500	1.000	1.000	2.000	3.000	3.000
AAD	0.250	0.333	0.500	0.500	1.000	2.000	2.000
RSI	0.143	0.167	0.333	0.333	0.500	1.000	1.000
DA	0.143	0.167	0.333	0.333	0.500	1.000	1.000

Table S4. Pairwise Comparison Matrix Used for Class 2 Devices in the Class-Based AHP Scenario.

Criterion (Class 2)	PSR	DF	DC	UF	AAD	RSI	DA
PSR	1.000	1.000	1.000	2.000	2.000	2.000	3.000
DF	1.000	1.000	1.000	2.000	2.000	2.000	3.000
DC	1.000	1.000	1.000	2.000	2.000	2.000	3.000
UF	0.500	0.500	0.500	1.000	1.000	1.000	3.000
AAD	0.500	0.500	0.500	1.000	1.000	1.000	3.000
RSI	0.500	0.500	0.500	1.000	1.000	1.000	3.000
DA	0.333	0.333	0.333	0.333	0.333	0.333	1.000

Table S5. Pairwise Comparison Matrix Used for Class 3 Devices in the Class-Based AHP Scenario.

Criterion (Class 3)	PSR	DF	DC	UF	AAD	RSI	DA
PSR	1.000	1.000	1.000	2.000	2.000	3.000	3.000
DF	1.000	1.000	1.000	2.000	2.000	3.000	3.000
DC	1.000	1.000	1.000	2.000	2.000	3.000	3.000
UF	0.500	0.500	0.500	1.000	1.000	1.000	4.000
AAD	0.500	0.500	0.500	1.000	1.000	1.000	4.000
RSI	0.333	0.333	0.333	1.000	1.000	1.000	5.000
DA	0.333	0.333	0.333	0.250	0.250	0.200	1.000

Code S1. MATLAB Code for Fuzzy AHP Criterion Weight Calculation.

```
%% Fuzzy AHP Analysis Script for Medical Device Prioritization

% Written for MATLAB R2024a

% Reference: Buckley's Geometric Mean Method & Center of Area Defuzzification

clc; clear; close all;

%% 1. Linguistic Scale to Triangular Fuzzy Numbers (TFN) Mapping

% Format: [l, m, u]

% 1: (1, 1, 1), 3: (2, 3, 4), 5: (4, 5, 6), 7: (6, 7, 8), 9: (8, 9, 9)

% Reciprocals are handled automatically in the pairwise matrix construction.

%% 2. Input: Crisp Pairwise Comparison Matrix (from Expert Consensus)

% Criteria:

% 1: Patient Safety Risk (PSR)

% 2: Device Function (DF)

% 3: Device Criticality (DC)

% 4: Utilization Frequency (UF)

% 5: Availability of Alternative Devices (AAD)

% 6: Resource Impact (RSI)

% 7: Device Age (DA)

crisp_matrix = [

    1.000, 1.000, 1.000, 3.000, 4.000, 4.000, 7.000;

    1.000, 1.000, 1.000, 3.000, 3.000, 3.000, 6.000;
```

```
    1.000, 1.000, 1.000, 3.000, 3.000, 3.000, 6.000;
    0.333, 0.333, 0.333, 1.000, 1.000, 1.000, 2.000;
    0.250, 0.333, 0.333, 1.000, 1.000, 1.000, 2.000;
    0.250, 0.333, 0.333, 1.000, 1.000, 1.000, 2.000;
    0.143, 0.167, 0.167, 0.500, 0.500, 0.500, 1.000
];
n = size(crisp_matrix, 1);
%% 3. Fuzzification Function (Saaty Scale to TFN Conversion)
fuzzy_matrix = cell(n, n);
for i = 1:n
    for j = 1:n
        val = crisp_matrix(i, j);
        fuzzy_matrix{i, j} = crispToTFN(val);
    end
end
%% 4. Fuzzy AHP Calculations (Buckley's Geometric Mean Method)
% Step 4.1: Calculate geometric mean of each row's fuzzy values
row_geom_mean = cell(n, 1);
for i = 1:n
    l_prod = 1; m_prod = 1; u_prod = 1;
    for j = 1:n
        l_prod = l_prod * fuzzy_matrix{i, j}(1);
        m_prod = m_prod * fuzzy_matrix{i, j}(2);
        u_prod = u_prod * fuzzy_matrix{i, j}(3);
    end
    row_geom_mean{i} = [l_prod^(1/n), m_prod^(1/n), u_prod^(1/n)];
end
% Step 4.2: Sum of all row geometric means
sum_l = 0; sum_m = 0; sum_u = 0;
for i = 1:n
```

```
    sum_l = sum_l + row_geom_mean{i}(1);
    sum_m = sum_m + row_geom_mean{i}(2);
    sum_u = sum_u + row_geom_mean{i}(3);
end
% Reverse and sort for fuzzy weights summation power
inv_sum = [1/sum_u, 1/sum_m, 1/sum_l];
% Step 4.3: Calculate Fuzzy Weights (Wi = Ri o (R1 + R2 + ... + Rn)^-1)
fuzzy_weights = cell(n, 1);
for i = 1:n
    l_w = row_geom_mean{i}(1) * inv_sum(1);
    m_w = row_geom_mean{i}(2) * inv_sum(2);
    u_w = row_geom_mean{i}(3) * inv_sum(3);
    fuzzy_weights{i} = [l_w, m_w, u_w];
end

%% 5. Defuzzification (Center of Area / Centroid Method)
% Formula: Crisp_Weight = (l + m + u) / 3
defuzz_weights = zeros(n, 1);
for i = 1:n
    tfn = fuzzy_weights{i};
    defuzz_weights(i) = (tfn(1) + tfn(2) + tfn(3)) / 3;
end
%% 6. Normalization of Crisp Weights
final_weights = defuzz_weights / sum(defuzz_weights);
%% Display Results
criteria_names = {'Patient Safety Risk', 'Device Function', 'Device Criticality', ...
                'Utilization Frequency', 'Availability of Alternatives', ...
                'Resource Impact', 'Device Age'};
disp('----------------------------------------------------');
disp('          FUZZY AHP CRITERIA WEIGHTS RESULTS          ');
```

```
disp('--------------------------------------------------');
fprintf('%-30s | %-20s | %-10s\n', 'Criteria', 'Fuzzy Weight (l,m,u)', 'Crisp Weight');
disp('--------------------------------------------------------------------------------');
for i = 1:n
    tfn_str = sprintf('(%.4f, %.4f, %.4f)', fuzzy_weights{i}(1), fuzzy_weights{i}(2), fuzzy_weights{i}(3));
    fprintf('%-30s | %-20s | %.4f\n', criteria_names{i}, tfn_str, final_weights(i));
end
disp('--------------------------------------------------');
%% Helper Function: Crisp to TFN Conversion
function tfn = crispToTFN(val)
    % Approximate mapping for crisp values to Triangular Fuzzy Numbers
    if val >= 1
        % Standard Saaty values: 1, 3, 5, 7, 9 and intermediates
        l = max(1/9, val - 1);
        m = val;
        u = min(9, val + 1);

        % Fine-tuning standard mappings
        if val == 1
            tfn = [1, 1, 1];
        elseif val == 3
            tfn = [2, 3, 4];
        elseif val == 5
            tfn = [4, 5, 6];
        elseif val == 7
            tfn = [6, 7, 8];
        elseif val == 9
            tfn = [8, 9, 9];
        else
            tfn = [l, m, u];
```

```
        end
    else
        % Reciprocal values (e.g., 1/3, 1/5, etc.)
        orig = 1 / val;
        if abs(orig - 3) < 0.1
            tfn = [1/4, 1/3, 1/2];
        elseif abs(orig - 5) < 0.1
            tfn = [1/6, 1/5, 1/4];
        elseif abs(orig - 7) < 0.1
            tfn = [1/8, 1/7, 1/6];
        elseif abs(orig - 9) < 0.1
            tfn = [1/9, 1/8, 1/7];
        elseif abs(orig - 2) < 0.1
            tfn = [1/3, 1/2, 1];
        else
            tfn = [1/(val+1), val, 1/(max(0.1, val-1))]; % general fallback
        end
    end
end
```

Code S2. MATLAB Code for the Integrated Fuzzy AHP–Fuzzy TOPSIS Analysis.

```
%% Fuzzy TOPSIS Analysis Script for Medical Device Prioritization
% Written for MATLAB R2024a
% Integrates Fuzzy AHP weights and performs Fuzzy TOPSIS evaluation for 32 devices
clc; clear; close all;
%% 1. Data Input: 32 Devices x 7 Criteria (Raw Crisp Matrix 1-5)
% Criteria order: [PSR, DF, DC, UF, AAD, RSI, DA]
raw_decision_matrix = [
    5, 5, 5, 5, 2, 5, 1; % 1. IABP
    5, 5, 5, 5, 4, 3, 3; % 2. Mechanical Ventilator
```

5, 5, 5, 5, 4, 3, 3; % 3. External Defibrillator

5, 5, 5, 5, 2, 3, 3; % 4. Neonatal Incubator

5, 5, 5, 5, 4, 5, 4; % 5. External Cardiac Pacemaker

5, 5, 5, 5, 5, 5, 3; % 6. Anesthesia Machine

5, 5, 5, 5, 4, 3, 2; % 7. Dialysis/Hemofiltration Unit

5, 5, 5, 4, 4, 3, 3; % 8. Cardiopulmonary Bypass (CPB)

5, 5, 5, 3, 3, 4, 1; % 9. Extracorporeal Membrane Oxygenation

4, 4, 4, 2, 1, 1, 4; % 10. Infusion Pump

3, 5, 4, 5, 1, 5, 2; % 11. CT Scanner

3, 5, 4, 5, 1, 5, 2; % 12. MRI Scanner

3, 5, 4, 3, 1, 5, 1; % 13. Gamma Camera

3, 4, 3, 4, 3, 3, 2; % 14. Doppler Ultrasound System

4, 5, 5, 5, 5, 5, 4; % 15. ECG System

2, 3, 2, 4, 2, 1, 4; % 16. Fetal Monitor

3, 4, 3, 3, 2, 2, 3; % 17. Laboratory Autoanalyzer

1, 3, 2, 4, 4, 1, 4; % 18. Intensive Care Monitoring

1, 2, 1, 2, 4, 1, 4; % 19. Phototherapy Unit

3, 4, 3, 4, 2, 4, 3; % 20. ECHO System

1, 2, 1, 1, 5, 1, 5; % 21. General Patient Monitor

3, 3, 2, 3, 2, 4, 3; % 22. X-ray System

2, 4, 2, 2, 1, 1, 3; % 23. Sterilization Unit (Autoclave)

1, 2, 1, 3, 5, 1, 5; % 24. Examination Lamp

1, 4, 1, 2, 3, 2, 5; % 25. Patient Scale

1, 3, 1, 1, 1, 1, 3; % 26. Treadmill

1, 2, 2, 2, 1, 1, 1; % 27. Hematology Staining Device

1, 4, 3, 3, 1, 2, 2; % 28. Ultrasonic Cleaning Bath

1, 1, 1, 1, 1, 2, 1; % 29. Water Bath

5, 5, 5, 5, 4, 4, 4; % 30. Reusable ECG Cables

3, 4, 3, 4, 2, 3, 3; % 31. Dental Equipment

4, 5, 4, 3, 1, 2, 2 % 32. Medical Sterilizer

```
];
device_names = {
    'IABP', 'Mechanical Ventilator', 'External Defibrillator', 'Neonatal Incubator', ...
    'External Cardiac Pacemaker', 'Anesthesia Machine', 'Dialysis/Hemofiltration Unit', ...
    'Cardiopulmonary Bypass (CPB)', 'Extracorporeal Membrane Oxygenation', 'Infusion Pump', ...
    'CT Scanner', 'MRI Scanner', 'Gamma Camera', 'Doppler Ultrasound System', ...
    'ECG System', 'Fetal Monitor', 'Laboratory Autoanalyzer', 'Intensive Care Monitoring', ...
    'Phototherapy Unit', 'ECHO System', 'General Patient Monitor', 'X-ray System', ...
    'Sterilization Unit (Autoclave)', 'Examination Lamp', 'Patient Scale', 'Treadmill', ...
    'Hematology Staining Device', 'Ultrasonic Cleaning Bath', 'Water Bath', ...
    'Reusable ECG Cables', 'Dental Equipment', 'Medical Sterilizer'
};
[m_dev, n_crit] = size(raw_decision_matrix);
%% 2. Fuzzy AHP Criteria Weights (Obtained from Part A)
% Format: [l, m, u] for each of the 7 criteria
fuzzy_weights = [
    0.1573, 0.2614, 0.3402; % PSR
    0.1364, 0.2355, 0.3131; % DF
    0.1364, 0.2355, 0.3131; % DC
    0.0445, 0.0785, 0.1138; % UF
    0.0525, 0.0753, 0.1746; % AAD
    0.0525, 0.0753, 0.1746; % RSI
    0.0358, 0.0384, 0.1957  % DA
];
%% 3. Fuzzification of Decision Matrix (1-5 Scale to TFN Mapping)
fuzzy_decision_matrix = cell(m_dev, n_crit);
for i = 1:m_dev
    for j = 1:n_crit
        val = raw_decision_matrix(i, j);
        fuzzy_decision_matrix{i, j} = scaleToTFN(val);
```

```
    end
end
%% 4. Normalization of Fuzzy Decision Matrix
normalized_fuzzy_matrix = cell(m_dev, n_crit);
column_maxs = zeros(n_crit, 3);
% Find max upper bound (u) for each column (Benefit criteria)
for j = 1:n_crit
    max_u = 0;
    for i = 1:m_dev
        if fuzzy_decision_matrix{i, j}(3) > max_u
            max_u = fuzzy_decision_matrix{i, j}(3);
        end
    end
    column_maxs(j, :) = [max_u, max_u, max_u];
end
for i = 1:m_dev
    for j = 1:n_crit
        tfn = fuzzy_decision_matrix{i, j};
        mx = column_maxs(j, 1);
        normalized_fuzzy_matrix{i, j} = [tfn(1)/mx, tfn(2)/mx, tfn(3)/mx];
    end
end
%% 5. Weighted Normalized Fuzzy Decision Matrix
weighted_fuzzy_matrix = cell(m_dev, n_crit);
for i = 1:m_dev
    for j = 1:n_crit
        norm_tfn = normalized_fuzzy_matrix{i, j};
        w_tfn = fuzzy_weights(j, :);
        % Multiplication of two TFNs
```

```
        weighted_fuzzy_matrix{i, j} = [norm_tfn(1)*w_tfn(1), norm_tfn(2)*w_tfn(2),
norm_tfn(3)*w_tfn(3)];
    end
end
%% 6. FPIS and FNIS Determination
FPIS = cell(1, n_crit);
FNIS = cell(1, n_crit);
for j = 1:n_crit
    max_u = -inf;
    min_l = inf;
    for i = 1:m_dev
        tfn = weighted_fuzzy_matrix{i, j};
        if tfn(3) > max_u
            max_u = tfn(3);
        end
        if tfn(1) < min_l
            min_l = tfn(1);
        end
    end
    FPIS{j} = [max_u, max_u, max_u];
    FNIS{j} = [min_l, min_l, min_l];
end
%% 7. Distance Calculation and Closeness Coefficient (CC)
closeness_coefficients = zeros(m_dev, 1);
for i = 1:m_dev
    d_plus = 0;
    d_minus = 0;
    for j = 1:n_crit
        v_ij = weighted_fuzzy_matrix{i, j};
        fpis_j = FPIS{j};
```

```
        fnis_j = FNIS{j};

        d_plus = d_plus + sqrt(1/3 * ((v_ij(1)-fpis_j(1))^2 + (v_ij(2)-fpis_j(2))^2 + (v_ij(3)-fpis_j(3))^2));
        d_minus = d_minus + sqrt(1/3 * ((v_ij(1)-fnis_j(1))^2 + (v_ij(2)-fnis_j(2))^2 + (v_ij(3)-fnis_j(3))^2));
    end
    closeness_coefficients(i) = d_minus / (d_plus + d_minus);
end
%% 8. Ranking with Tie-Handling Rule
[sorted_cc, original_indices] = sort(closeness_coefficients, 'descend');
%% Display Final Results
disp('------------------------------------------------------------------------');
disp('                FUZZY TOPSIS MEDICAL DEVICE PRIORITIZATION              ');
disp('------------------------------------------------------------------------');
fprintf('%-5s | %-35s | %-12s\n', 'Rank', 'Medical Device', 'Closeness CC');
disp('------------------------------------------------------------------------');
for r = 1:m_dev
    idx = original_indices(r);
    fprintf('%-5d | %-35s | %.4f\n', r, device_names{idx}, sorted_cc(r));
end
disp('------------------------------------------------------------------------');
%% Helper Function: Linguistic Scale to TFN Mapping
function tfn = scaleToTFN(score)
    switch score
        case 1
            tfn = [1, 1, 2];
        case 2
            tfn = [1, 2, 3];
        case 3
            tfn = [2, 3, 4];
        case 4
```

```
            tfn = [3, 4, 5];
        case 5
            tfn = [4, 5, 5];
        otherwise
            tfn = [1, 1, 1];
    end
end
```
